# Combining machine learning and high-throughput experimentation to discover photocatalytically active organic molecules†

**DOI:** 10.1039/d1sc02150h

**Published:** 2021-06-21

**Authors:** Xiaobo Li, Phillip M. Maffettone, Yu Che, Tao Liu, Linjiang Chen, Andrew I. Cooper

**Affiliations:** Department of Chemistry & Materials Innovation Factory, University of Liverpool 51 Oxford Street Liverpool L7 3NY UK xiaobo.li@liverpool.ac.uk lchen@liverpool.ac.uk aicooper@liverpool.ac.uk; National Synchrotron Light Source II, Brookhaven National Laboratory Upton New York 11973 USA; Leverhulme Research Centre for Functional Materials Design, Materials Innovation Factory and Department of Chemistry, University of Liverpool 51 Oxford Street Liverpool L7 3NY UK

## Abstract

Light-absorbing organic molecules are useful components in photocatalysts, but it is difficult to formulate reliable structure–property design rules. More than 100 million unique chemical compounds are documented in the PubChem database, and a significant sub-set of these are π-conjugated, light-absorbing molecules that might in principle act as photocatalysts. Nature has used natural selection to evolve photosynthetic assemblies; by contrast, our ability to navigate the enormous potential search space of organic photocatalysts in the laboratory is limited. Here, we integrate experiment, computation, and machine learning to address this challenge. A library of 572 aromatic organic molecules was assembled with diverse compositions and structures, selected on the basis of availability in our laboratory, rather than more sophisticated criteria. This training library was then assessed experimentally for sacrificial photocatalytic hydrogen evolution using a high-throughput, automated method. Quantum chemical calculations and machine learning were used to visualise, interpret, and ultimately to predict the photocatalytic activities of these molecules, covering a much broader chemical space than for previous polymer photocatalyst libraries. By applying unsupervised learning to the molecular structures, we identified structural features that were common in molecules with high catalytic activity. Further analysis using calculated molecular descriptors within a suite of supervised classification algorithms revealed that light absorption, exciton electron affinity, electron affinity, exciton binding energy, and singlet–triplet energy gap had correlations with the photocatalytic performance. These trained predictive models can be used in future studies as filters to deprioritise or discard would-be low-activity candidate molecules from experiments, and to prioritize more favourable candidates. As a demonstration, we used virtual *in silico* experiments to show that it was possible to halve the experimental cost of finding 50% of the most active photocatalysts by using the machine learning model as an experimental advisor. We further showed that the ML advisor trained on the 572-molecule library could be used to make predictions for an unseen set of 96 molecules, achieving equivalent predictive accuracies to those in the initial training set. This marks a step toward the machine-learning assisted discovery of molecular organic photocatalysts and the approach might also be applied to problems beyond photocatalytic hydrogen evolution, such as CO_2_ reduction and photoredox chemistry.

## Abstract 2

We developed models to predict the photoactivity of organic molecules for photocatalytic hydrogen evolution by integrating experiment, computation, and machine learning. This marks a step toward the data-driven discovery of molecular photocatalysts.

## Graphical abstract

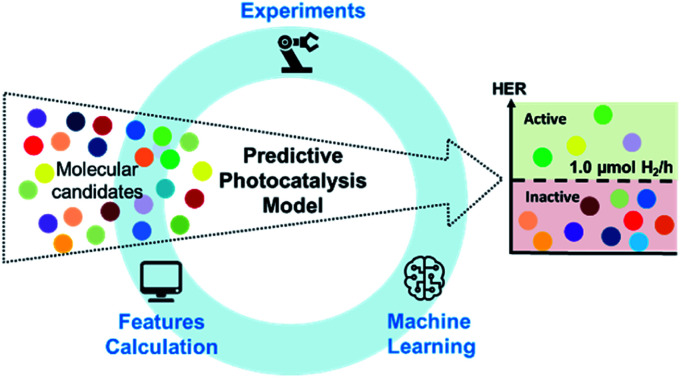

## Introduction

Conjugated organic materials such as carbon nitride, conjugated linear polymers, conjugated organic frameworks, and π-conjugated molecules have emerged as photocatalysts for solar fuels generation^1–6^ and for photoredox organic synthesis.^7–10^ However, it remains challenging to predict the activity of organic photocatalysts, either by expert knowledge or by using *a priori* computations. This is because catalytic performance is influenced by a host of factors spanning multiple length scales, such as light absorption, thermodynamic driving force, exciton recombination, charge carrier mobility, physical surface properties, and so on. Beyond the basic question of whether a given material is likely to absorb visible light, these factors are generally hard to predict. Also, the variables interact in complex ways:^11^ for example, porosity might be desirable to increase the catalyst surface area, but it might also reduce charge carrier mobility. To deconvolute such multivariate relationships, we need algorithms to model multi-dimensional datasets. We also need a sufficient volume of data to create meaningful models. At present, most studies in the literature are focused on a handful of catalysts, making it difficult to probe general structure–activity relationships. To complicate things further, photocatalytic activity is critically dependent on the precise details of the experimental set up, such as the intensity of the light source: as such, notwithstanding the surge of interest and publications in this area, the lack of experimental standardisation between laboratories makes it challenging to implement data-mining approaches across a large number of different studies. We need larger and more standardized datasets to have a chance of learning the underlying structure–property rules.

Machine learning techniques are used increasingly to complement experiments when studying complex chemical systems, often in combination with quantum chemical calculations or molecular modelling, as exemplified by the accelerated discovery of drugs,^12^ catalysts,^11,13–17^ porous adsorbents,^18^ and batteries.^19^ A key goal is to generate both predictive models and to gain physical insights: the first allows for fast, *in silico* pre-evaluation of potential candidates through quantitative prediction, qualitative ranking, or coarse-grained filtering; the second allows us to understand the relationship between material structure and physicochemical characteristics and its functional properties. Machine learning strategies are sometimes also referred to as “data-driven”, with the implication that they require sizable datasets to train on. This limits the power of such methods when the acquisition of experimental data is time-consuming and expensive.

To our knowledge, the largest library of organic photocatalysts that was experimentally tested under identical conditions contained 175 conjugated linear polymers.^11^ All of those polymers were alternating AB co-polymers, where one of the two comonomers already known to promote photocatalytic activity. Here, we set out to create a larger and more diverse library of candidate organic molecules that could be rapidly sourced and tested for sacrificial photocatalytic hydrogen evolution as a training set. This strategy has several advantages. First, π-conjugated organic molecules are a promising class of photocatalysts: various aromatic compounds such as benzophenone,^20^ proflavin,^21^ and dyes (boron-dipyrromethene,^22^ xanthene,^23,24^*etc.*^25,26^), have been studied for photocatalytic hydrogen evolution,^27,28^ but there is a far larger space of π-conjugated molecules that are as yet unstudied. Second, in contrast with polymer photocatalysts, which are mixtures of molecules with a wide range of molar masses, organic molecules are attractive choices for data-driven studies because of their unambiguous molecular structures, defined composition, and chemical purity. Third, no new synthesis was involved in this study: we worked with molecules that were already available in our laboratory based on historical research activity. This allowed us to rapidly expand the size of the library to include 572 molecules. The size of this library could be increased further in the future, while preserving the data consistency required for applying pre-trained machine-learning models to new data. We focused only on aromatic molecules here, based on the assumption that the presence of a π-conjugated system is necessary for light absorption in the spectral range of the light source used in this study (350–1000 nm). The second criterion was availability in our laboratory. As such, the library is diverse, containing many molecules that were originally sourced or synthesized for other applications, such as the synthesis of porous organic cages, conjugated microporous polymers, and covalent organic frameworks, as well as some molecules that had been explored in photocatalysis-related problems; for example, as potential sensitizers, monomers for polymers, or precursors for monomers, *etc.* Apart from aromaticity and availability, no other prior knowledge about the desirable properties of the candidate photocatalysts was applied in the library selection, thus minimising prior chemical knowledge from skewing the structure–activity correlation. As a result of this broad selection approach, we expected the hit rate for good photocatalysts to be modest.

With a large dataset of organic photocatalyst activities in hand, we then carried out density functional theory calculations and machine learning to visualise, interpret, and predict the activity of the molecules. By applying unsupervised learning to atomic neighbour environments, we identified correlations between molecular structure and photocatalytic activity that is to some extent human interpretable. These structure–activity correlations were further demonstrated to be machine learnable for predicting the photocatalytic activity. We also used a suite of supervised classification algorithms, together with calculated molecular descriptors, to construct predictive models for hydrogen evolution rates, which reveals key optoelectronic properties that impact the performance of these molecular photocatalysts. Finally, the potential of machine-learning driven advisors to assist chemists in the discovery of new photocatalysts was illustrated by *in silico* virtual experiments and experimental blind tests.

## Results and discussions

### A library of candidate organic photocatalysts

**Fig. 1:**
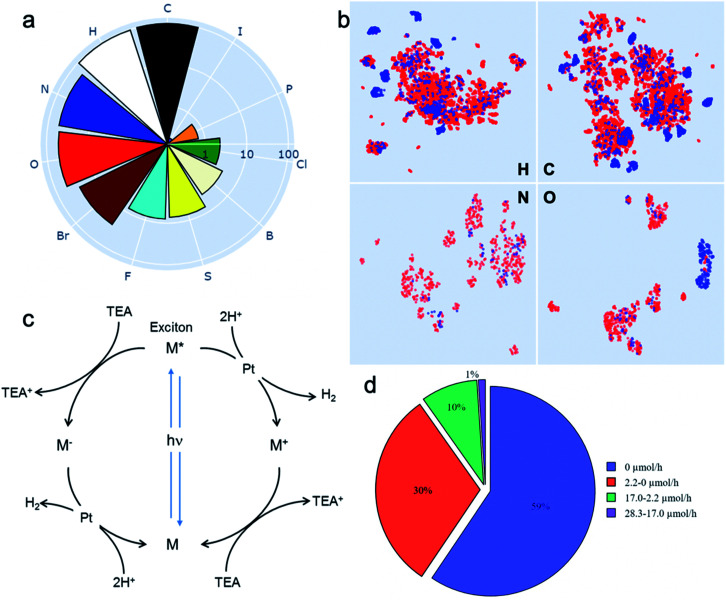
A library of 572 aromatic candidate organic photocatalyst molecules. (a) Polar bar chart showing the percentage of molecules in the library containing the 11 different chemical elements that occur: 100% contain C, 96% contain H, and so on. The radial coordinates are on a logarithmic scale. (b) Comparison of the diversity of atomic neighbour environments for H, C, N and O elements found in this molecular library (red points) and in the library of 99 conjugated polymers reported in ref. 11 (blue points). The molecular library covers a significantly larger chemical space. (c) Scheme showing the proposed photocatalytic processes involved in sacrificial hydrogen production. M = organic molecule. Conditions used here: 5 mg molecular catalyst, triethylamine/MEOH/H_2_O (1 : 1 : 1 vol%) mixture, 3 wt% Pt (formed *in situ*), solar simulator irradiation (spectral range of source: 350–1000 nm). (d) Statistical summary of the photocatalytic hydrogen evolution performance of the candidate molecular catalysts in the library. The hydrogen evolution rate (HER) was classified against two conjugated polymers as a benchmark: carbon nitride PCN^29^ (2.2 μmol h^−1^) and a covalent triazine framework CTF-1 (ref. 30) (17.0 μmol h^−1^).

Drawing on our laboratory's existing chemical stocks, we identified 572 aromatic molecules and investigated their performance for photocatalytic hydrogen evolution activity. No selection bias other than aromaticity and availability in our laboratory was applied. A total of 11 elements occurred in this library of molecules; the frequencies of their occurrence is shown in Fig. 1a. To help visualise the structures of all the 572 molecules, we also developed an interactive browser-based explorer for our library (https://www.molecular-photocatalysts-library.app).

To assess the chemical diversity of our molecules in terms of the chemical space coverage, we compared them with the linear polymer photocatalysts reported by Bai *et al.*,^11^ which is the largest library of organic photocatalysts in a single study to date. We used the Smooth Overlap of Atomic Positions (SOAP)^31^ descriptor to encode atomic neighbour environments for the H, C, N and O elements in both libraries. For visual comparison, we applied the Uniform Manifold Approximation and Projection (UMAP) technique^32^ to learn a mapping from the high-dimensional SOAP vectors to a two-dimensional (2D) representation (Fig. 1b). This showed that our library of 572 molecules covers a significantly larger chemical space than the polymer library,^11^ as expressed by the four key elemental types (C, H, O and N; Fig. 1B). The higher chemical diversity of our molecular library stems from the larger number of occurring elements (11 *vs.* 8), as well as our library containing a significantly larger number of different molecules than the total number of unique monomers in the polymer library (572 *vs.* 99). Also, that polymer library was constructed by using chemical knowledge: specifically, it was biased to include comonomers such as dibenzosulfone that were already known to promote photocatalytic activity.^33^

**Fig. 2:**
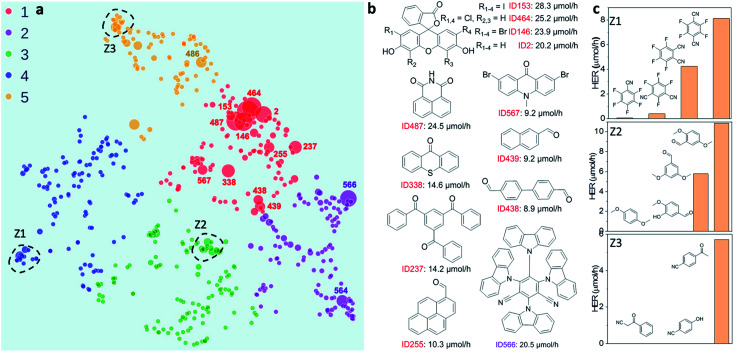
Structure–activity map of the molecular photocatalyst library. (a) 2D UMAP embedding of the chemical space of the photocatalyst library, colour-coded by *k*-means clusters identified using the 2D UMAP coordinates; symbol size denotes the experimentally measured hydrogen evolution rate (HER). (b) Molecules selected based on their high HERs; their locations on the UMAP plot are labelled in (a). (c) Plots showing relative HERs for groups of structurally similar molecules; the locations of these three groups (Z1–Z3) are circled on the UMAP plot in (a).

The photocatalytic hydrogen evolution performance for the small molecule library was investigated using a high-throughput parallel photocatalysis screening platform that utilizes a solar simulator, as described previously.^11^ Triethylamine (TEA) and Pt (produced by *in situ* photoreduction) were used as the sacrificial agent and as a proton reduction catalyst, respectively. For molecular organic photocatalysts (and to a great extent conjugated polymers), the exciton binding energy is large relative to *kT* (26 meV at room temperature). Hence, spontaneous dissociation of excitons into free electrons and holes is difficult. The generalised catalytic mechanism proposed here is that the photo-generated excitons on the molecule can either undergo a single-electron reduction or oxidation, mediated by the sacrificial electron donor (TEA) and the proton reduction catalyst (Pt), respectively (Fig. 1c). Fig. 1d shows a statistical summary of the photocatalytic hydrogen evolution rates (HERs) of the dataset. In comparison with two benchmark conjugated polymers PCN^29^ and CTF-1,^30^ synthesised in-house and measured under exactly the same conditions, 63 molecules showed HERs higher than for PCN (2.2 μmol h^−1^), and 6 molecules surpassed the HER for CTF-1 (17.0 μmol h^−1^). The highest HER among our molecular photocatalysts (ID153; see Fig. 2b for structure) was 28.3 μmol h^−1^ (5660 μmol g^−1^ h^−1^), which is comparable to the highest HER (∼6000 μmol g^−1^ h^−1^) measured for the 175 conjugated polymers using the same experimental setup using a more design-led approach.^11^ A full list of the molecules (ID1-ID572) is provided in the ESI.†

### Mapping structure–activity correlations

To investigate possible structure–activity correlations in our library, we used SOAP descriptors to encode the molecules and, together with a regularised entropy match (REMatch) kernel,^34^ to quantify the similarity between all pairs of molecules. The resulting similarity matrix was then projected onto a 2D space by a UMAP embedding, as shown in Fig. 2a, where each point represents a molecule. The size of each point relates to the photocatalytic activity of the molecule (the HER). The points are arranged spatially such that the closer the two points are on the plot, the more similar the two molecules are, as described by SOAP. We further used the *k*-means algorithm to identify clusters on the 2D UMAP space, showing that the 572 molecules can be broadly clustered into five groups, colour coded in Fig. 2a, based on their chemical and structural similarity.

Fig. 2a shows that there are correlations between molecular structure and hydrogen evolution activity in the dataset (symbol size scaled by measured HER). For example, the molecules in the library with high HERs are mostly located in group 1 (red points on plot). Within each of the five larger sub-groupings, molecules with relatively high catalytic performance tend to form smaller, local clusters. Structural analysis of the molecules with the highest activities (>9 μmol h^−1^) revealed that all but one examples (ID566) shared the common structural feature of having at least one aryl carbonyl moiety (Fig. 2b, and S1a†). However, it is worth noting that molecules with similar structures can show large differences in hydrogen evolution activities; for example, the structural isomers shown in sub-cluster Z1, Fig. 2c.

### Descriptors for molecular photocatalysts

For an organic molecule to act as an efficient hydrogen evolution photocatalyst according to the scheme in Fig. 1c, it must absorb light efficiently and drive thermodynamically the reduction of protons and the oxidation of water or, in this study, a sacrificial agent (TEA). To achieve this, the electron affinity (EA) of the molecule or its exciton ionization potential (IP*) and the ionization potential (IP) of the molecule or its exciton electron affinity (EA*) must straddle the proton reduction and the TEA oxidation potentials. The exciton potentials (EA* and IP*) are important for molecular photocatalysts, where the exciton binding energy (*E*_eb_)—that is, the energy binding an electron–hole pair through the electrostatic Coulomb force—is generally large and spontaneous dissociation of excitons into free electrons and holes is difficult. We used density functional theory (DFT) and time-dependent (TD) DFT calculations to determine these energy levels computationally; details are given in the Methods section and the ESI.†

We also performed detailed analyses of a range of key optoelectronic and excited-state properties, that is: (i) light absorption (optical gap, Δ*E*_S_1_→S_0__), (ii) excited-state charge distribution (change in dipole moment between S_1_ and S_0_, Δ*D*; degree of spatial extension of hole and electron distribution in the charge-transfer direction, *H*_CT_) and charge separation (difference in the extent of spatial distribution between electron and hole, Δ*σ*; electron–hole overlap, *S*_r_), and; (iii) the energy gap between the first singlet (S_1_) state and the first triplet (T_1_) state (Δ*E*_S_1_→T_1__). We also calculated the solvation energy (*E*_sol_) of the molecule in water as a potential indicator of its wettability, as well as the self-binding (in a dimer) energy, *E*_b_, of the molecule as a proxy for its propensity for aggregating with itself. Full definitions of these descriptors and calculation details are given in the ESI.†

### Machine learning the hydrogen evolution activity

**Fig. 3:**
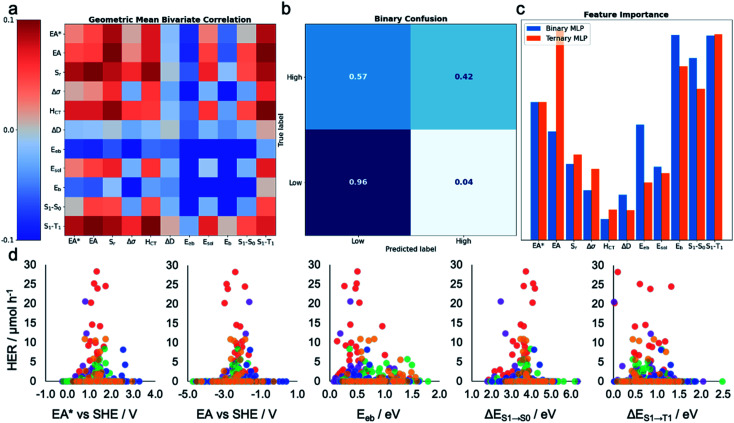
Machine learning the activity of the molecular photocatalysts. (a) Bivariate Pearson's correlation between the hydrogen evolution rate (HER) and all pairs of the calculated molecular descriptors, where the scale runs between −0.1 and +0.1; the diagonal running from the top-left corner to the bottom-right corner shows the correlation between the HER and individual descriptors. (b) Confusion matrix for the MLP-based binary classifier. (c) Extracted permutation feature importance for the MLP models in binary and ternary classification tasks. (d) Measured HERs of all the 572 molecules plotted against the five key molecular features suggested by the MLP models; SHE: standard hydrogen electrode. The symbols are colour-coded by the *k*-means clusters as shown in Fig. 2a.

To gain insight into the dependence of HER on these various calculated descriptors, we first explored the Pearson's correlation coefficients for individual features and their binary combinations. The cells on the diagonal (top-left to bottom-right) of Fig. 3a show the extent of linear correlation of the HER with individual variables, while the off-diagonal cells contain the geometric mean of the correlation of HER with each of the two descriptors. The absolute value of the Pearson correlation coefficient is less than 0.1 for all variables and variable pairs, indicating a weak linear correlation, if any, between the HER and single descriptors or binary combinations of them. This shallow statistical analysis will not capture any complicated or nonlinear behaviours dependent on multiple features, but it confirms that any possible structure–property–activity relationship in our dataset is of a nonlinear, multivariate nature. Indeed, Fig. S4† shows that the HERs are not linearly dependent on any individual descriptors, nor is any pair of the calculated molecular descriptors correlated in a simple way.

Next, a number of machine learning (ML) models were evaluated for their suitability to construct predictive models together with the computed molecular descriptors. This included *k*-nearest neighbours (KNN), random forests (RF), support vector machines (SVM), Gaussian processes (GP), gradient boosted decision trees (GB-DT), and multilayer perceptrons (MLP), all of which have been used in various areas of chemistry and materials science.^35–37^ The models were trained for tiered classification tasks based on optimised HER thresholds. By transforming a regression problem into a lower-resolution classification problem, the models act as a filtration step for flagging potentially photoactive candidate molecules. For binary classification, this resulted in one class being assigned to HER values smaller than 1.07 μmol h^−1^, with the other class assigned to values larger than 1.07 μmol h^−1^. The class thresholds (in μmol h^−1^) for ternary classification were 1.07 and 12.5; that is, low: HER ≤ 1.07, medium: 1.07 < HER ≤ 12.5, high: HER > 12.5. The quaternary classification was also attempted, as well as regression tasks, but no satisfactorily predictive models could be achieved.

Leave-one-out results showed that the calculated molecular descriptors were successful at producing binary and ternary classifications with greater than 87% accuracy, independent of the model type (Table 1). The use of 10-fold cross-validation affords computational efficiency but fails to produce high *F*1-scores. This was a result of class imbalance: there are far more data points in the ‘low’ performance class than in the ‘high’ performance class (492 *vs.* 80), and 59% of molecules in the library produced no hydrogen at all, thus exposing the classifier to more information related to the low-performance case. This occurs when the dataset is sampled uniformly for each fold of cross validation; this issue remains when using biased sampling to force each fold to have a constant amount of each class. Class imbalance is a core challenge in applying machine learning to a wide range of research problems in the physical sciences, such as diversity-oriented screening for new photocatalysts, where there are often far more zeros in a dataset than nonzero values. Overall, our results show that the use of molecular descriptors that quantify a range of photochemical and electronic features of the molecule, in conjunction with ML models, can predictively assign HER performance levels (low, medium or high) to candidate photocatalysts, albeit with limitations.

**Table 1:** Binary and ternary classification metrics across all models, obtained by 10-fold and leave-one-out (LOO) cross-validation procedures^a^

Model	Binary				Ternary		
	10-fold		LOO accuracy	CM MCC^d^	10-fold		LOO accuracy
	Accuracy^b^	*F*1-score^c^			Accuracy	*F*1-score	
KNN	0.89	0.69	0.89	0.46	0.89	0.61	0.89
GP	0.87	0.57	0.87	0.30	0.87	0.42	0.87
RF	0.89	0.69	0.88	0.35	0.88	0.57	0.89
GB-DT	0.89	0.73	0.89	0.44	0.88	0.57	0.89
SVM	0.87	0.68	0.87	0.34	0.88	0.58	0.87
MLP	0.89	0.71	0.89	0.46	0.89	0.56	0.88

a Analyses of the area under the curve (AUC) for the receiver operating characteristic (ROC) curve and the precision-recall (PR) curve for all the binary-classification models are given in Table S2.

b The sum of the number of true positives (TP) and true negatives (TN) divided by the sum of the number of true positives, true negatives, false positives (FP), and false negatives (FN).

c Weighted harmonic mean of precision and recall, where precision is the number of true positives divided by the sum of the number of true positives and false positives; recall is the number of true positives divided by the number of true positives and false negatives. For ternary classification, metrics are computed independently for each class and then averaged (macro average).

d The Matthews correlation coefficient (MCC), calculated directly from the binary confusion matrix (CM; Fig. S12–S17), as 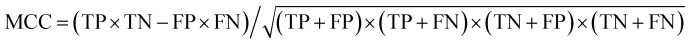, ranging between −1 and 1; values greater than zero indicate a performance better than random assignment.

Comparing KNN with the other ML models (Tables 1 and S2†), we note the relative similarity of the models and the largely interpolative behaviour of them, with featurization being more important than the ML model itself. This is unsurprising because of the class imbalance challenge described above as well as missing any mesoscale experimental factors, which are not captured by the current set of molecular descriptors, as discussed further below.

To assess the practical utility of these models, we explored when and how they failed. From the confusion matrices shown in Fig. 3b and S12–S17,† it is clear that the experimentally high-performing catalysts are more often mislabelled as being ‘low’ performers than the opposite case. This is a result of the significant class imbalance, discussed above. The current models are robust against producing false positives (more than 95% ‘low’ performers are correctly labelled by all the models; Fig. S12–S17†), and hence useful to screen out candidates that would show zero or low hydrogen evolution activities. Some ‘high’ performing molecules will also be eliminated because they are mislabelled as ‘low’ performing, but this behaviour could be acceptable when the cost of experiment is high and evaluating an excess of candidates becomes expensive—for example, to guide investigations that cannot access high-throughput screening facilities, as used here. To minimise the model's proneness to false negatives—that is, mislabelling ‘high’ performers as ‘low’—more data points in the ‘high’ HER class would be required to improve the model's performance. By examining these confusion matrices and the performance metrics in Table 1, we identified the MLP models as the strongest binary and ternary classifiers.

Binary and ternary classification tasks were also performed for the 572 molecules, using only the molecular structure as input representation (Section 3.5, ESI†). To encode the molecules for machine learning, both Morgan fingerprints and SOAP descriptors were tested, together with using the Tanimoto index or the REMatch kernel as the similarity measure (further details are given in Table S3†). KNN and SVM models were evaluated for both structural representations, using their respective, precomputed distance metrics. All the KNN models outperformed their SVM counterparts, in both binary and ternary classifications, for both structural representations (Table S3 and Fig. S24–S27†). The SOAP-based KNN model was identified as the strongest binary classifier, while the Morgan fingerprints-based KNN model was identified as the strongest ternary classifier; both of these models performed well in both binary and ternary classification tasks. These results show that the structure–activity correlations that are only somewhat human-interpretable in Fig. 2a are machine-learnable, achieving equivalent levels of predictive ability to the strongest ML models using engineered descriptors (*cf.*Table 1). It would be particularly advantageous to use structure-based ML models to guide large-scale experimental screening of molecular photocatalysts, as expensive descriptor calculations would otherwise become the bottleneck to increasing the throughput. Naturally, such models do not intuitively highlight physical features of high-performance photocatalysts, which could then be used to guide the design of better catalysts, nor do they reveal directly the structural features that may have correlated with the photocatalytic activity.

### Understanding the important molecular features for photocatalytic activity

Beyond their predictive ability, interpretability is a key goal for ML models to understand the importance of each descriptor and to obtain physical insights into structure–property–activity relationships. Permutation importance was calculated for all of the models presented in Table 1 and these are shown in Fig. 3c and S18–S23.† The MLP models, the strongest binary and ternary classifiers, assign high relative importance to exciton electron affinity (EA*), electron affinity (EA), exciton binding energy (*E*_eb_), optical gap (Δ*E*_S_1_→S_0__), and singlet–triplet energy gap (Δ*E*_S_1_→T_1__) for both binary and ternary classification tasks. EA* estimates the thermodynamic driving force for the molecular photocatalyst to oxidise the sacrificial agent, TEA. EA estimates the thermodynamic driving force for proton reduction. Δ*E*_S_1_→S_0__ estimates the optical gap of the molecular photocatalyst. EA*, EA and Δ*E*_S_1_→S_0__ are intuitively important, because they are essential optoelectronic requirements for a molecule to act as a photocatalyst: that is, the molecule must absorb light efficiently over a broad range in the visible spectrum as well as having enough thermodynamic driving force to oxidize TEA (EA*) or to reduce protons (EA). Importantly, our ML models identified two additional molecular properties, *E*_eb_ and Δ*E*_S_1_→T_1__, that correlate strongly with a high photocatalytic activity. *E*_eb_ represents the energy penalty for separating an exciton into free charges and large values of *E*_eb_ would be expected to lead to fast exciton recombination, thus unfavourably influencing the photocatalytic activity. Δ*E*_S_1_→T_1__ is the energy gap between the first singlet state and the first triplet state; the smaller the value of Δ*E*_S_1_→T_1__ is, the larger the spin–orbital coupling, the faster the rate of intersystem crossing and, ultimately, the higher the triplet exciton yield. Fig. 3d shows that the high-activity (>12.5 μmol h^−1^) molecules tend to have a small *E*_eb_ and a small Δ*E*_S_1_→T_1__.

Molecules ID146, ID153, ID255, ID338 and ID487 are among the most active photocatalysts in this study and share the common structural feature of having at least one aryl carbonyl moiety (Fig. 2a). Their high HERs might be attributed, at least in part, to their ability to generate triplet excitons.^24,31,38^ More generally, many aryl carbonyl compounds, such as ketones and aldehydes, are known for their high yields of excited triplet states.^39–43^ Molecule ID566 has a high HER of 20.5 μmol h^−1^ and is known for its thermally activated delayed fluorescence (TADF),^44^ a process that entails the molecule harvesting triplet excitons. In general, triplet excitons have a longer lifetime than singlet excitons, which may be beneficial for allowing the photo-generated excitons on the molecule to participate in redox processes with the sacrificial agent and/or the Pt cocatalyst, rather than undergoing recombination to the ground state.

Both the feature importance analyses of the trained ML models (Fig. 3c and d) and the structural analyses of the most active photocatalysts in our library (Fig. 2) highlighted a positive correlation between the formation of triplet excitons and high hydrogen evolution activity. For the future design of molecular photocatalysts, an effective strategy may be to boost the formation of triplet excitons by incorporating auxochrome moieties (such as the carbonyl group) and/or heavy atoms (such as I, Br, and S) into the molecular structure, as exemplified by some selected pairs in our library shown in Fig. S1.† However, an attempt to correlate HERs for some of the molecules with their reported triplet-state yields in isolation failed to produce any correlation, as shown in Fig. S2.† This is perhaps unsurprising since the hydrogen evolution activity of a photocatalyst is rarely governed by a single physicochemical or optoelectronic property but rather by a host of molecular and mesoscale factors. As such, more sophisticated approaches—such as the structure-based or the descriptor-based machine learning demonstrated here—hold the promise for using data-driven strategies to probe the complex structure–property–activity relationship for molecular photocatalysts.

In addition to the intrinsic challenge of classifying reactions that are dictated by a complex, interrelated set of factors, there are other experimental factors that may contribute to the difficulty of this classification task. First, while we conducted all reactions under the same experimental conditions, the generalised mechanism proposed in Fig. 1c makes various assumptions: for example, we assume that the hydrogen produced is generated from the water, rather than from the organic molecule itself. Usually, we would confirm this for each reaction, for example *via* isotopic labelling experiments, but this is more challenging for such a large library of reactions. Some molecules in our library—such as ID67, ID146, ID153, and ID566—were already reported by others to perform photocatalytic hydrogen evolution in the presence of sacrificial agents.^20,38,45^ Also, molecules ID98, ID182 and ID183 show measured HERs of 2.6, 4.2 and 8.1 μmol h^−1^, respectively, but contain no H atoms; hence, the H_2_ produced cannot come from the organic molecule itself. Another consideration is solubility: while the molecules selected have, on the whole, low aqueous solubilities, some molecules in the library might have finite solubility in the water/TEA/MEOH mixture, and we did not try to account for this in the descriptors. Also, the interaction between the organic molecules and the Pt cocatalyst is important—which could be influenced by particle size, surface properties, or the Pt loading method—but these factors were not captured explicitly by any of the descriptors used. That said, the objective of this work was to build a useful classifier with affordable experimental cost, and in this respect, a balance must be struck between exactness and complexity of the experiments. In this regard, it took us only 15 working days to explore the 572-member molecular library using the high-throughput screening platform that we developed.^11^ This platform can perform 48 samples per day (1 hour to weigh samples; 6 hours for N_2_ purge and liquid dispense (overnight); 3 hours for light illumination, and a further 8 hours for GC analysis).

### Virtual experiments and blind tests

**Fig. 4:**
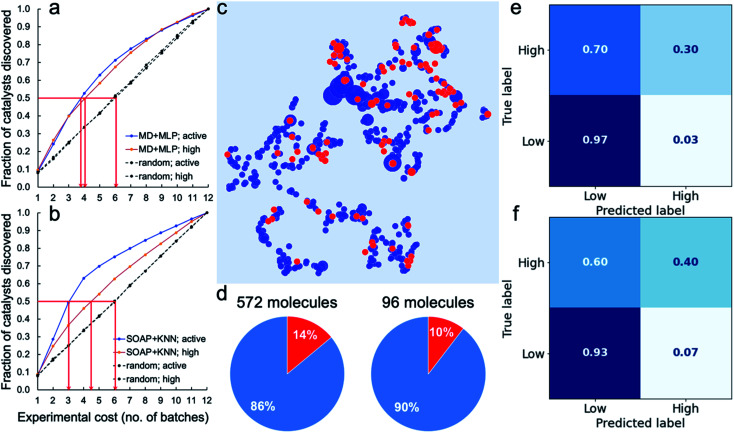
Virtual experiments and blind tests. (a and b) Virtual experiments comparing an adaptive machine learning approach with random sampling: the 572 molecules were encoded by the molecular descriptors (MD; the same as in Fig. 3) and trained with MLP models (a) or encoded by the SOAP descriptors (the same as in Fig. 2a) and trained with KNN models (b). Active samples were defined as having HERs > 1.07 μmol h^−1^ and high-activity samples as having HERs > 12.5 μmol h^−1^. The average number of batches taken to find 50% of the active and highly active catalysts is marked by the red arrows. A total of 200 *in silico* experiments was carried out for both the ML approach and for the random sampling method, each with a different random starting point, to obtain these average results. (c–f) Blind tests of the ML models trained on the 572 molecules (referred to as the 572-molecule library) for 96 unseen molecules (referred to as the blind-test set). (c) 2D UMAP embedding of the chemical space (encoded by SOAP) of the 572-molecule library (in blue) and the blind-test set (in red); the symbol size is scaled by the experimentally measure HER. (d) Percentages (in red) of the active samples (HERs > 1.07 μmol h^−1^) in the 572-molecule library and the blind-test set. (e and f) Confusion matrices for the predictions of the blind-test set by models based on the MD + MLP protocol (e) or the SOAP + KNN protocol (f), both trained on the 572-molecule library.

To assess the potential for using an ML ‘advisor’ to discover molecular photocatalysts, we carried out *in silico* experiments on the 572 molecules using their measured HERs as the ground truth to evaluate the search performance. To do this, we compared the use of an adaptive ML advisor with random sampling (Fig. 4a). In these *in silico* virtual experiments, 48 samples were ‘measured’ in each batch, which matches the batch-size of the real high-throughput experiments. In the ML advisor approach, an MLP binary classifier and an MLP ternary classifier were trained on all known data after each batch, and then used to predict a class for each of the remaining untested molecules. The next batch was then chosen from the untested molecules until the 48 slots were filled by selecting, in the following priority order: (i) molecules predicted by the ternary classifier to have high-activity (HER > 12.5 μmol h^−1^); (ii) molecules predicted by both the ternary classifier and the binary classifier to be active (HER > 1.07 μmol h^−1^); and (iii) molecules predicted by the binary classifier to be active. When necessary, the batch of 48 molecules was completed with molecules selected randomly from the non-active class. The classification models were then rebuilt after each batch. For the random sampling approach, each batch of 48 molecules was simply chosen randomly from the untested molecule pool. Fig. 4a shows that it took, on average, about 3.8 and 4.0 batches to discover 50% of the active and high-activity catalysts, respectively, using the ML advisor. Using the random selection approach, it took 6 batches to discover the same proportion of active and highly active photocatalysts. Similarly, using binary and ternary classifiers both built on SOAP-based KNN models (Fig. 4b), the adaptive approach was able to discover 50% of the active and high-activity catalysts within, on average, about 3.0 and 4.5 batches, respectively. The use of this adaptive ML advisor to assist the chemist could therefore significantly reduce the experimental cost for finding promising photocatalysts, thus providing a predictive method to explore large molecular search spaces.

We further tested our ML advisor on 96 molecules that were not included in the initial 572-molecule photocatalyst library, to better assess its potential in real-world applications. The 96 molecules, referred to as the blind-test set, were selected considering only their aromaticity and (again) availability in our lab, as for the first 572 molecules. They were measured in two batches using our high-throughput parallel photocatalysis screening platform. The blind-test set falls within the chemical space of the 572-molecule library (Fig. 4c) and has a similar percentage (10%) of active samples to that of the 572-molecule library (14%; Fig. 4d) — 10 out of the 96 molecules had HERs larger than 1.07 μmol h^−1^, none of which was greater than 12.5 μmol h^−1^. In predicting for the blind-test set, the MLP model was again identified as the strongest binary classifier, when combined with the calculated molecular descriptors (Fig. 4e, and S29–S34†); the MLP model was ranked second for ternary classification, with a slightly inferior performance to the KNN model. Binary classification for the blind-test samples directly from their molecular structures (Fig. 4f), encoded by SOAP descriptors, using KNN yielded an equivalent level of predictive accuracy to that achieved by the strongest binary classifier using molecular descriptors. For ternary classification, the descriptor-based KNN markedly outperformed the structure-based KNN (Fig. S34 and S35†). These blind-test results confirmed the potential of using an ML advisor to assist the chemists in the discovery of new molecular photocatalysts, as well as highlighting the promise for structure-based ML models to facilitate large-scale high-throughput screening.

Looking forward, the predictive ability of machine learning for molecular photocatalysis might be improved by capturing additional information for the higher-activity molecules. For example, efficient charge transfer between the molecular photocatalyst and the sacrificial agent or the co-catalyst is key to catalytic performance, but such intermolecular effects are not considered explicitly here. Future work in engineering descriptors might focus on better capturing the charge-transfer characteristics of the system, as well as the exciton lifetime and transport properties. Second, populating the dataset in the high-activity region is essential for training robust, predictive machine-learning models. Our initial results suggest hypothesis-led strategies, such as investigating molecules that are known for their thermally activated delayed fluorescence,^46^ room temperature phosphorescence,^47^ or photoinduced singlet oxygen production,^48^ all of which necessitate the formation of excited triplet states. We further expect that model assembling might increase the robustness of these approaches. Ensembles should include models trained against a variety of descriptors, including those derived from the molecular structure and those abstracted from graph neural networks,^49^ for example. Transfer learning may be a particularly promising strategy because the acquisition of large experimental datasets can be time-consuming and expensive; here, models are pre-trained on large datasets with relevant or surrogate properties, followed by task-specific fine-tuning for predictive modelling. The experimental study presented here was a single batch process; that is, all of the experiments were done prior to model building. This was done because it was tractable to attempt measuring HERs for all 572 molecules in the available library using the high-throughput automated methods that we have developed.^11^ For much larger libraries, or where such automation is not available, a more efficient approach would be to build the model ‘on the fly’, as in the virtual experiments above, and to recommend the next batch of molecules as the model evolves. This could also tackle the class imbalance problem that is discussed above. In this respect, a closed-loop autonomous search would be particularly attractive.^50–52^

## Conclusions

Molecular photocatalysts are a promising and relatively less explored avenue for hydrogen production, as well as other photocatalytic transformations such as CO_2_ reduction and hydrogen peroxide synthesis,^53,54^ but the potential chemical space is enormous, and most of this space remains unexplored. We assembled here the largest library of organic photocatalysts tested experimentally to date and tested all 572 molecules under identical experimental conditions using a high-throughput testing methodology. We further tested 96 molecules as a blind-test set for evaluating the trained ML models, bring the combined total of experimentally measured molecules to 668. This is a tiny fraction of the total available chemical space, but large enough to construct useful ML structure–property–activity models. We used unsupervised learning and supervised classification to reveal the structural features and optoelectronic properties that positively impact the activity of these molecular photocatalysts for sacrificial hydrogen production, which also allowed some physical interpretations: for example, the formation of triplet excitons seems to have a beneficial effect. This suggests further exploration of molecules known for intersystem crossing. Despite being sourced simply on the basis of availability in our laboratory, rather than any more sophisticated rationale, ∼1% of the molecules in the library (5 in total) performed comparably (4040–5660 μmol g^−1^ h^−1^) to the highest HER (∼6000 μmol g^−1^ h^−1^) measured for the 175 conjugated polymers using the same experimental setup from a more design-led but much more synthetically expensive approach.^11^ Moreover, some of the active molecules that were discovered—for example, 1,8-naphthalimide (ID487), tetrafluoroterephthalonitrile (ID183), and 1,3,5-tribenzoylbenzene (ID237)—have not to our knowledge been explored before in this context.

Virtual experiments show that an adaptive ML-assisted selection approach outperforms random sampling (Fig. 4a and b), significantly reducing the experimental cost of identifying the active photocatalysts in the library. A further evaluation of the trained ML advisor on a blind test set of 96 molecules confirmed its potential in assisting the discovery of new molecular photocatalysts. We did not benchmark our methods against expert knowledge: for example, where a chemist pre-sorts the library in terms of probable photocatalytic activity. We note, however, that while some of the active catalysts discovered could have been prioritized based on existing literature reports (*e.g.*, ID67, ID146, ID153, and ID566),^20,38,45^ others were unknown and—to us at least—non-intuitive, such as ID183 and ID237. As such, these fast screening methods can create new inspiration for future research directions. We would expect the ML-assisted rapid screening method to be particularly helpful for problems where there is little or no prior literature to draw upon—for example, in the search for photocatalysts that illicit new, unknown reactivity in organic transformations, where the initial hit rate will be low by definition.

This dataset of molecular structures, measured photocatalytic activities, and calculated molecular descriptors joins other established, public databases of organic molecules^55–57^ and offers a test-bed for groups interested in new machine learning methods. Also, the online interactive explorer that we developed here (https://www.molecular-photocatalysts-library.app) might allow for new physical insights and/or new design principles to be developed by other catalysis researchers. In summary, this is one of a relatively small number of studies where machine learning methods have been integrated with high-throughput property measurements across a sizeable and diverse set of materials (668 organic molecules in total). This makes an important step towards accelerating the discovery of molecular photocatalysts and allows us to consider a much broader chemical space than we have contemplated so far.

## Methods

### Library selection

We sourced the 572 molecules on the basis of (i) the presence of an aromatic unit and (ii) their availability in our laboratory on the scale needed for experimental testing. The same selection criteria were adopted when sourcing a further set of 96 molecules for blind tests of the machine-learning models.

### High-throughput hydrogen evolution experiments

Agilent Technologies vials (10 mL) were charged with 5.0 ± 0.1 mg of the organic molecule to be tested and transferred to a Chemspeed Accelerator SWING workstation for liquid transfer. Degassed jars with triethylamine, methanol, and a stock solution of H_2_PtCl_6_ were loaded into this automated liquid handling platform. The system was then closed, and the entire cabinet was purged for 4 h with nitrogen. The automated liquid handling platform then dispensed the liquids as follows: (i) degassed aqueous H_2_PtCl_6_ solution (1.7 mL, 3 wt% Pt w. r. t. to the organic molecule); (ii) triethylamine (1.7 mL), and (iii) methanol (1.7 mL). The pH of the solution was typically around 11.5. The vials were then capped using the capper/crimper tool on the platform under inert conditions. Once capped, the samples were removed, shaken, and transferred to an ultrasonic bath to disperse the photocatalysts, which were typically insoluble (by eye) in the reaction medium. Oriel Solar Simulator 94123A with an output of 1.0 sun was used to illuminate the vials on a Stuart roller bar SRT9 for the time specified (classification IEC 60904-9 2007 spectral match A, uniformity classification A, temporal stability A, 1600 W Xenon light source, 12 × 12 in.2 output beam, air mass 1.5 G filter, 350–1000 nm). After photocatalysis, the gaseous products of the samples were measured on an Agilent gas connected to a headspace sampler (HS) and Shimadzu GC-HS. No hydrogen evolution was observed for mixtures of water/triethylamine/methanol or water/triethylamine/methanol/H_2_PtCl_6_ under the identical conditions. For a subset of 89 molecules, HER measurements were repeated at least twice. Results for the 28 top-performing molecules are shown in Fig. S3,† confirming that the HER measurements were reproducible; other molecules in this sub-set had low (<1.0 μmol h^−1^) or zero HERs with high repeatability.

To investigate the effect of the Pt cocatalyst loading on the photoactivity of these molecular photocatalysts, six structurally diverse molecules with varying levels of photocatalytic performance were studied with different Pt loadings, ranging from 0 to 5 wt%. Fig. S36† plots the HERs of these six molecules as a function of the Pt loading. The presence of the Pt cocatalyst is essential to achieve measurable HERs; none of the six catalysts showed an appreciable HER at zero Pt loading. The Pt loading also had no influence on the activity ranking of these six molecules. Some molecules, such as ID487 and ID146, showed lower HERs at Pt loadings above 1 wt%, probably because of light occlusion by excess photodeposited metal. Overall, while Pt loading is clearly important, the HER ranking of these six molecules is independent of the metal loading, at least in the loading range 1–5 wt%, though the results do show that comparisons should be made at equivalent cocatalyst loadings.

Suitable sacrificial agents are necessary for photocatalysts to exhibit hydrogen evolution activity. Common sacrificial agents include alcohols, amines, organic acids, sulfides/sulphites, and so on. Fig. S37† plots the HERs of the same six molecules measured with three different sacrificial agents: trimethylamine, methanol and ascorbic acid. The results show that trimethylamine is the most suitable sacrificial agent for these organic molecular photocatalysts.

### Computational and machine learning details

All DFT and TD-DFT calculations made use of the B3LYP density functional, together with the 6-31G* basis set (SDD was used for iodine atoms), using the Gaussian 16 software.^58^ Benchmarking of our choice of the level of theory using available experimental data and against the range-separated CAM-B3LYP functional, together with a larger basis set (Def2-SVP), is given in section 3.2 of the ESI (Fig. S5–S8†). The effect of solvation by water was accounted for by using the PCM/SMD solvation model. All (TD-)DFT calculations were based on the geometry optimised in the charge-neutral, ground state (at the same level of theory)—that is, vertical ionization and vertical excitation—except for the singlet–triplet energy gap (Δ*E*_S_1_→T_1__). For Δ*E*_S_1_→T_1__, the effect of excited-state relaxation on both the *S*_1_ and *T*_1_ potential energy surfaces was accounted for by optimizing the geometry in the respective state with TD-DFT employing the Tamm–Dancoff approximation. All electron excitation analyses were performed using Multiwfn.^59^

The Uniform Manifold Approximation and Projection (UMAP) technique was used for dimensionality reduction for mapping high-dimensional data to 2D representations, while preserving both global and local topological structures of the data in the high-dimensional space as much as possible. For Fig. 1b, we used all atoms of one of the four elemental types to learn the 2D UMAP embedding of their atomic neighbour environments, in which atoms of any elemental types may be present. In the resulting UMAP-learned 2D representation, points will overlay in the 2D space if they are at the same position in the original high-dimensional space.

All machine-learning models were implemented using the scikit-learn package^60^ except for the MLPs, which were implemented in PyTorch.^61^ Hyperparameters were optimised using a discrete Bayesian optimization^50^ and the scikit-optimise package.^62^ During model training and optimization, the dataset was split between 80% training and 20% test across 10 different folds. The target metric—accuracy and *F*1-score for classification—of the resulting 10 models is averaged across all folds during hyperparameter optimization. We then trained the best models under a leave-one-out dataset split. This is equivalent to *k*-folds, where *k* is the number of points in the dataset, and results in a convergence of accuracy and *F*1-score by definition. Table 1 summarises the optimal results obtained across all models, while the full optimised hyperparameters are given in Table S1.†

## Data availability

The authors declare that data supporting the findings of this study are available within the paper and its ESI.† The computational data that were used for machine learning in this study are included as ESI† and available from the online visualisation application.

## Author contributions

X. L., L. C. and A. I. C. conceived and supervised the project. X. L. carried out all the experimental work. P. M. M. and Y. C. performed the machine learning studies. Y. C. developed and deployed the online application for interactive data visualization. T. L. performed all the descriptor calculations. L. C. conceived the computational and machine-learning strategies and supervised their implementations. All authors interpreted the data; L. C., X. L. and A. I. C. led the preparation of the manuscript.

## Conflicts of interest

There are no conflicts to declare.

## Supplementary Material

SC-012-D1SC02150H-s001

SC-012-D1SC02150H-s002

SC-012-D1SC02150H-s003

SC-012-D1SC02150H-s004

SC-012-D1SC02150H-s005

SC-012-D1SC02150H-s006
