# Supplementary material for: Combining machine learning and high-throughput experimentation to discover photocatalytically active organic molecules
Source: Chem Sci. 2021 Jun 21;12(32):10742–54. doi: 10.1039/d1sc02150h (PMC8372320; doi:10.1039/d1sc02150h)
Supplement: SC-012-D1SC02150H-s004 [file SC-012-D1SC02150H-s004.pdf]

Electronic Supplementary Material (ESI) for Chemical Science.
This journal is © The Royal Society of Chemistry 2021

Electronic Supplementary Information

Combining machine learning and high-throughput experimentation to discover photocatalytically active organic molecules

Xiaobo Li,[a,†,*] Phillip M. Maffettone,[a,b,†] Yu Che,[a,c,†] Tao Liu,[a,†] Linjiang Chen,[a,c,*] Andrew I. Cooper[a,c,*]

[a] Department of Chemistry & Materials Innovation Factory, University of Liverpool, 51 Oxford Street, Liverpool L7 3NY, UK
[b] National Synchrotron Light Source II, Brookhaven National Laboratory, Upton, New York 11973, USA
[c] Leverhulme Research Centre for Functional Materials Design
† These authors contributed equally. * Corresponding authors

Table of Contents

1. General methods ***3***

2. Photocatalytic tests ***3***

3. Computational details ***6***

3.1. Structure generation **6**

3.2. Molecular descriptors **6**

3.3. The chemical space of the photocatalyst library as encoded by different representations.11

3.4. Machine learning with molecular descriptors **14**

3.5. Machine learning with molecular fingerprints and SOAP descriptors **21**

4. Virtual experiments with the 572 molecules ***24***

5. Blind tests for 96 molecules, unseen by the models trained on the 572-molecule library .25

5.1. Molecules encoded by molecular optoelectronic descriptors **25**

5.2. Molecules encoded by SOAP descriptors **28**

6. Experimental investigation of the effects of the amount of Pt cocatalyst and the choice of sacrificial agent on HERs ***29***

7. References ***30***

1. General methods

Small organic molecules, other reagents, and solvents were purchased from commercial suppliers (Manchester Organics, Sigma Aldrich, Alfa Aesar, Fluorochem, Ark Pharm, Apollo, Combi-Blocks, TCI Europe, Carbosynth, TRC Canada, etc.) and used with no further purification. PCN[1] and CTF[2] were prepared using previous methods. Water for the hydrogen evolution experiments was purified using an ELGA LabWater system with a Purelab Option S filtration and ion exchange column (ρ = 15 MΩ cm) without pH level adjustment.

2. Photocatalytic tests

High-Throughput Hydrogen Evolution Experiments. Agilent Technologies vials (10 mL) were charged with 5.0 ± 0.1 mg of small molecules and transferred to a Chemspeed Accelerator SWING robot for liquid transfer. Degassed jars with triethylamine, methanol, and a stock solution of H_2PtCl_6 were loaded into the automated liquid handling platform. The system was then closed and purged for 4 h with nitrogen. The automated liquid handling platform then dispensed the liquids as specified, which were degassed aqueous H_2PtCl_6 solution (1.7 mL, 3wt % Pt to small molecules), triethylamine (1.7 mL), and methanol (1.7 mL). The pH of the solution was around 11.5. The vials were then capped using the capper/crimper tool under inert conditions. Once capped, the samples were taken out, shaken briefly, and transferred to an ultrasonic bath to disperse the photocatalysts. An Oriel Solar Simulator 94123A with an output of 1.0 sun was then used to illuminate the vials on a Stuart roller bar SRT9 for the time specified (classification IEC 60904-9 2007 spectral match A, uniformity classification A, temporal stability A, 1600 W xenon light source, 12 × 12 in.2 output beam, air mass 1.5 G filter, 350−1000 nm). After photocatalysis, the gaseous products of the samples were measured on an Agilent gas connected to a headspace sampler (HS) and Shimadzu GC-HS. No hydrogen evolution was observed for mixtures of water/triethylamine/methanol or water/triethylamine/methanol/H_2PtCl_6 under the identical conditions.

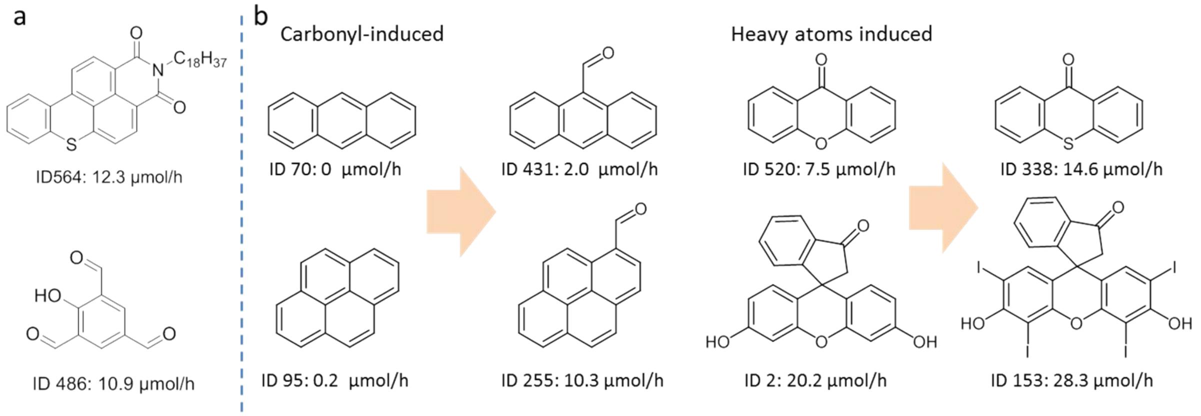

Figure S1. (a) Molecular structures for the selected molecules. (b) Selected examples of molecular photocatalysts showing improved hydrogen evolution after incorporating carbonyl groups or heavy atoms; both functionalities are known to facilitate the formation of triplet excited states, although it should be noted that the introduction of these moieties will affect other photophysical properties, too.

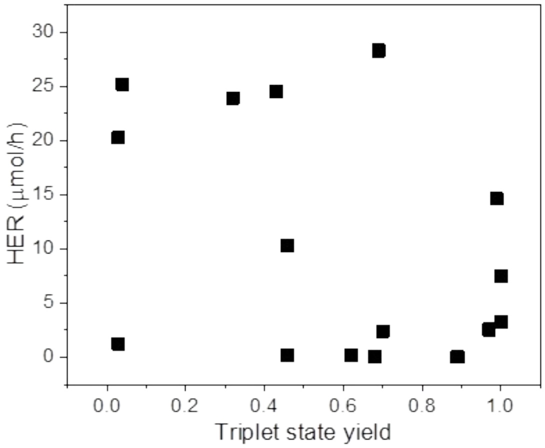

Figure S2. Hydrogen evolution rates (HERs) plotted as the function of their corresponding yields of the triplet states for a selection of the molecules in the library, as measured experimentally and reported in the literature. The raw data for making this plot and the relevant references are given in the supporting spreadsheet file.

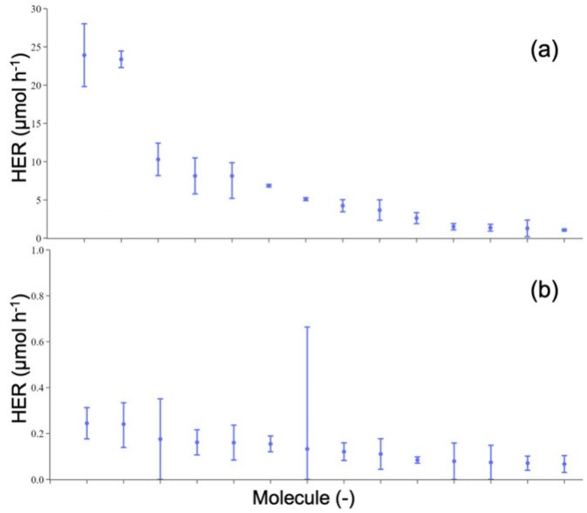

Figure S3. HER measurement repeats for a selected subset of molecules: (a) 14 molecules with HERs spanning the whole activity range covered by the complete library of 572 molecules; (b) 14 molecules with HERs below 1 μmol h^{-1}. For each molecule, the average HER from multiple (at least 2) repeats are shown, together with error bars indicating the maximum value and the minimum value among the repeats.

3. Computational details

3.1. Structure generation

To generate atomistic structures for the molecules, the simplified molecular-input line-entry system (SMILES) representation of each molecule was converted to a 3D structure using Open Babel.[3]The gen3d operation was used, which starts with 250 steps of steepest-descent geometry optimization with the MMFF94 forcefield, followed by 200 iterations of a Weighted Rotor conformational search, before a final 250-step conjugated-gradient geometry optimization. The resulting 3D structure was subjected to a further conformer search, generating 50 conformers. The lowest-energy conformer from the search was finally geometry-optimized at the B3LYP/6-31G* level of theory; this structure was then used as the starting geometry for the molecule in all following computational and machine-learning studies.

3.2. Molecular descriptors

A total of 13 descriptors were calculated for all of the 572 molecules: IP, EA, EA*, IP*, S_r, $\Delta\sigma$, H_{CT}, ΔD, E_{eb}, E_{sol}, E_b, $\Delta E_{S1\rightarrow S0}$, and $\Delta E_{S1\rightarrow T1}$.

IP, EA, EA* and IP* are standard reduction potentials of half-reactions for free electrons/holes and excitons and were calculated using (TD-)DFT (see Methods section for details). The exciton binding energy, E_{eb}, was calculated as E_{eb} = IP - EA* or E_{eb} = IP* - EA. It is clear that IP and IP* are related to EA* and EA, respectively, through E_{eb}. Therefore, only EA, EA* and E_{eb} were included in the descriptor vectors for machine learning.

S_r, $\Delta\sigma$, H_{CT}, and ΔD are descriptors from quantitative characterization of hole and electron distributions in real space, performed for the first singlet (S_1) state on the optimized, ground-state geometry, using the Multiwfn software. Briefly:

S_r index quantifies the overlap between the hole distribution ($\rho^{hole}(\boldsymbol{r})$) and the electron distribution ($\rho^{electron}(\boldsymbol{r})$). S_r varies between 0 (no overlap) and 1 (complete overlap); the larger the value is, the greater the extent of overlap is.

$\Delta\sigma$ index is the difference between $\boldsymbol{\sigma}_{electron}$ and $\boldsymbol{\sigma}_{hole}$, given by

$\Delta\sigma$ index = $| \boldsymbol{\sigma}_{electron} | - | \boldsymbol{\sigma}_{hole} |$,

where $\boldsymbol{\sigma}_{\text{electron}}$ and $\boldsymbol{\sigma}_{\text{hole}}$ are a measure of the sparsity of $\rho^{\text{electron}}(\boldsymbol{r})$ and $\rho^{\text{hole}}(\boldsymbol{r})$, respectively. $\Delta\sigma$ index can be positive or negative for different molecules.

H_{CT} is the average of $\boldsymbol{\sigma}_{\text{electron}}$ and $\boldsymbol{\sigma}_{\text{hole}}$ in the charge-transfer (CT) direction, given by

$H_{CT} = |\ \mathbf{H} \cdot \mathbf{u}_{CT}\ |$,

where $\mathbf{H} = (\boldsymbol{\sigma}_{\text{electron}} + \boldsymbol{\sigma}_{\text{hole}})/2$ and $\mathbf{u}_{CT}$ is the unit vector along the CT direction.

ΔD is the difference in dipole moment between the excited-state and the ground-state of the molecule—*i.e.*, $\Delta D = D_{\text{excited-state}} - D_{\text{ground-state}}$—a measure of the extent of charge redistribution between the two states.

$\Delta E_{S1\to S0}$ is the energy difference between the S_1 state and the S_0 state. $\Delta E_{S1\to S0}$ is also referred to as the calculated optical gap in this study.

$\Delta E_{S1\to T1}$ is the energy difference between the S_1 state and the first triplet (T_1) state. The smaller the $\Delta E_{S1\to T1}$ value, the larger the spin-orbital coupling, and ultimately the more probable and faster the intersystem crossing.

E_{sol} is an approximation of the solvation energy, simply given by

$E_{sol} = E_{solvated} - E_{gas}$,

where $E_{solvated}$ and E_{gas} are the total energy of the molecule in solvation (water, PCM/SMD) and gas phase, respectively. The molecular geometry was relaxed in each state.

E_b is the binding energy between two molecules of the same identity—intended as an indicator of the molecule's propensity for aggregating—which is given by

$E_{binding} = E_{dimer} - 2\times E_{monomer}$,

where E_{dimer} and $E_{monomer}$ are the total energy of the dimer and that of an isolated molecule, respectively. Stable binding conformations of a particular dimer were searched by a grid-based approach, together with the Amber forcefield, as implemented in the Autodock software.[4] The most stable dimer from the Autodock search was then geometry-optimized using the GFN-xTB semiempirical tight binding method,[5] with implicit water solvation. The isolated molecule in water solvation was geometry-optimized using the same computational settings.

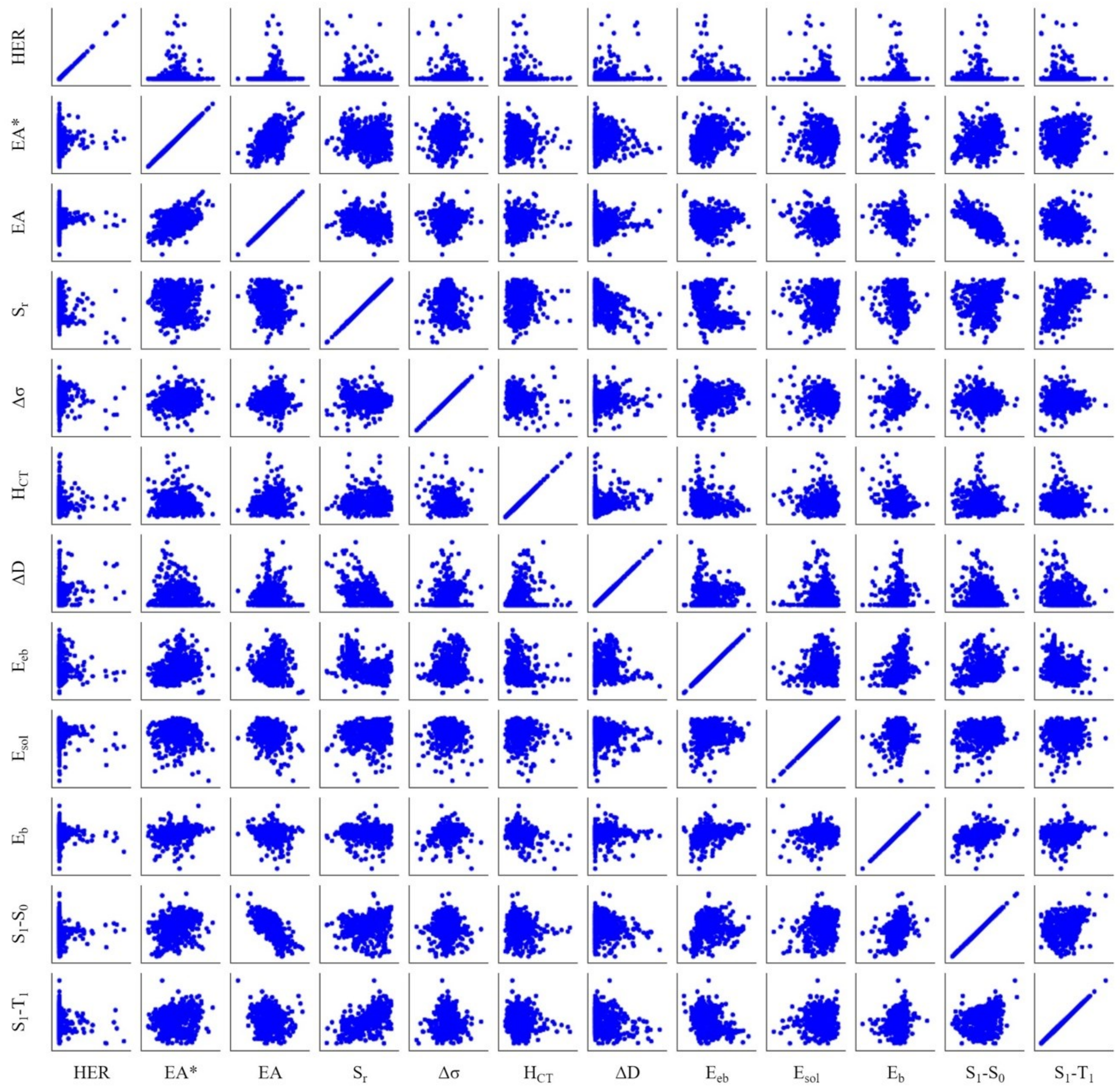

Figure S4. One-to-one correlation between all pairs of the calculated molecular descriptors and the measured HER: HER in μmol h^{-1}; EA* and EA in eV; S_r in a.u.; Δσ and H_{CT} in Å; ΔD in a.u.; E_{eb}, E_{sol}, E_b, $\Delta E_{S1\rightarrow S0}$ and $\Delta E_{S1\rightarrow T1}$ in eV.

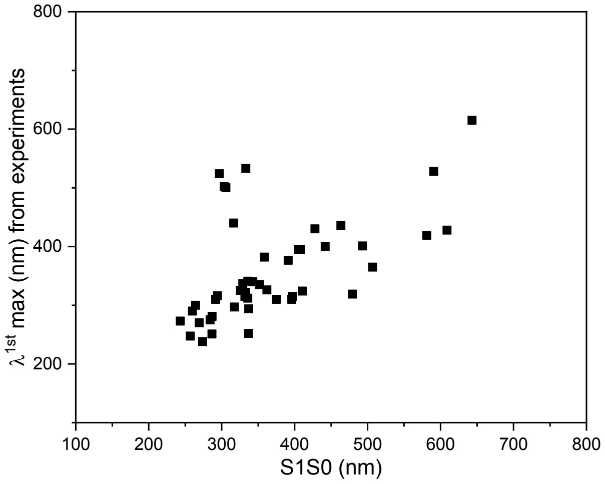

Figure S5. Comparison of TD-DFT calculated optical gap ($\Delta E_{S1\rightarrow S0}$) with experimental UV-vis λ_{max}^{1st} data for a selected subset of 46 molecules. The raw data and source references are available in the supporting spreadsheet file.

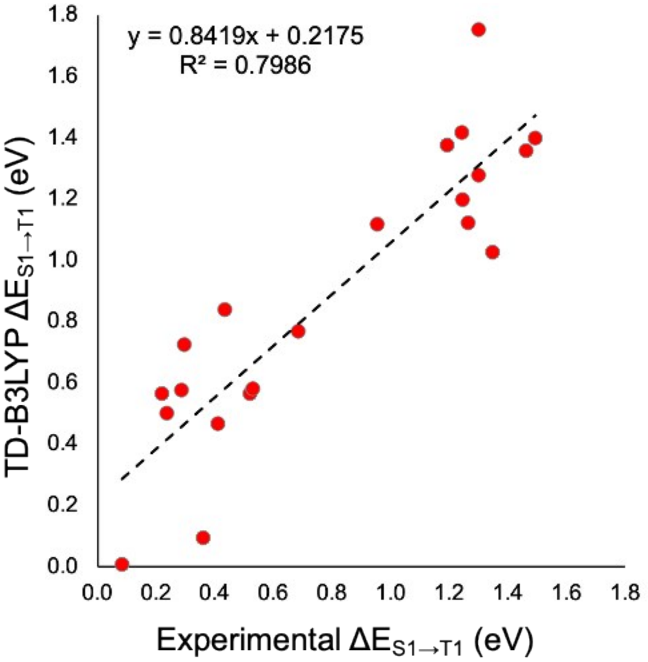

Figure S6. Comparison of TD-B3LYP-calculated and experimental $\Delta E_{S1\rightarrow T1}$ values for 22 out of the 572 molecules studied in this work. The dashed line indicates a linear fit to the data, with the resulting equation and goodness of fit displayed.

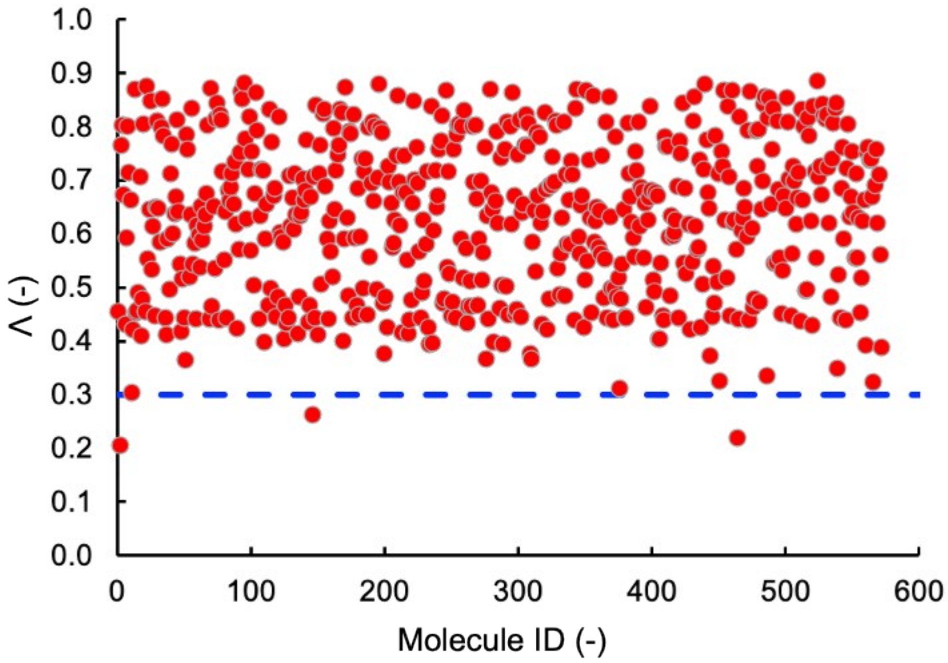

Figure S7. Calculated values of the Λ diagnostic tests[6] for the vertical S_1 states of all 572 molecules at the B3LYP/6-31G* level of theory. The blue, dashed line indicates the Λ value of 0.3, an empirical threshold below which potential TD-DFT charge-transfer problems may be expected.

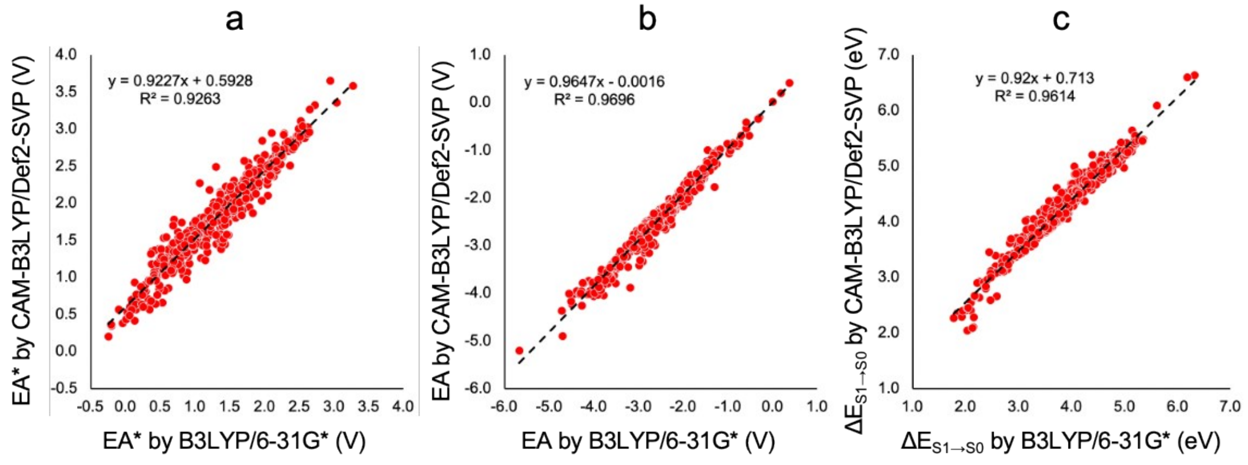

Figure S8. Comparison of EA* (a), EA (b) and $\Delta E_{S1\rightarrow S0}$ (c) of all 572 molecules using different levels of theory: (TD-)B3LYP/6-31G* or (TD-)CAM-B3LYP/Def2-SVP.

3.3. The chemical space of the photocatalyst library as encoded by different representations

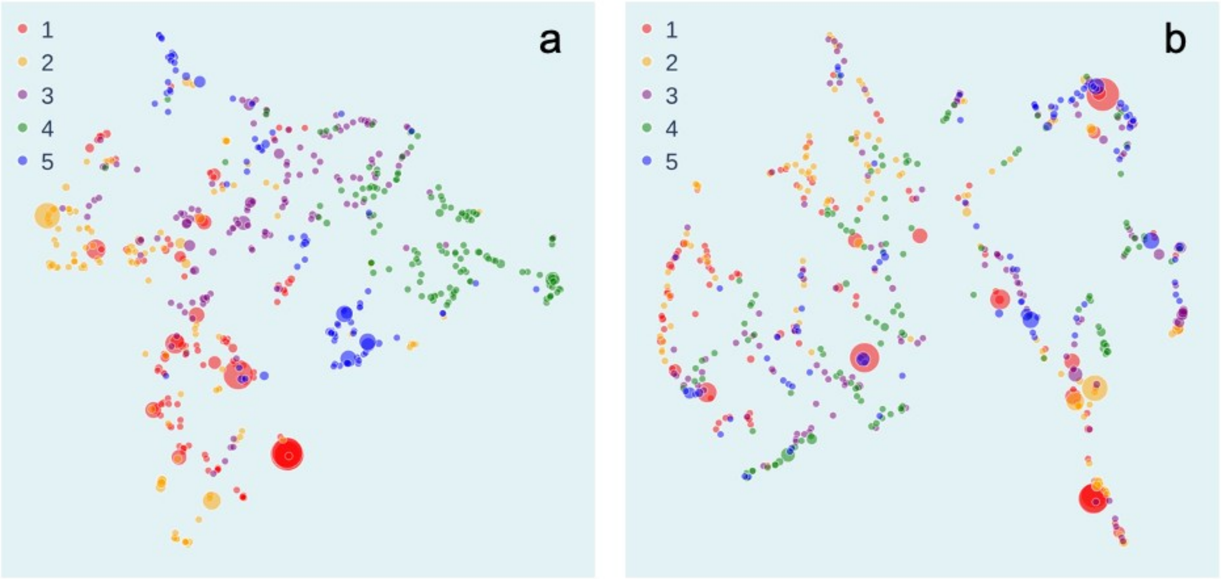

Figure S9. 2D UMAP embedding of the chemical space of the photocatalyst library, as defined by Morgan fingerprints, together with Tanimoto index as the similarity measure, in (**a**) or defined by the (TD)-DFT-calculated molecular descriptors, together with Euclidean distance as the similarity measure, in (**b**). Symbol size denotes the experimental hydrogen evolution rate; symbol colour denotes the k-means cluster as shown in Figure 2a in the main text, where the chemical space of the library is defined by the SOAP descriptors, together with a REMatch kernel as the similarity measure.

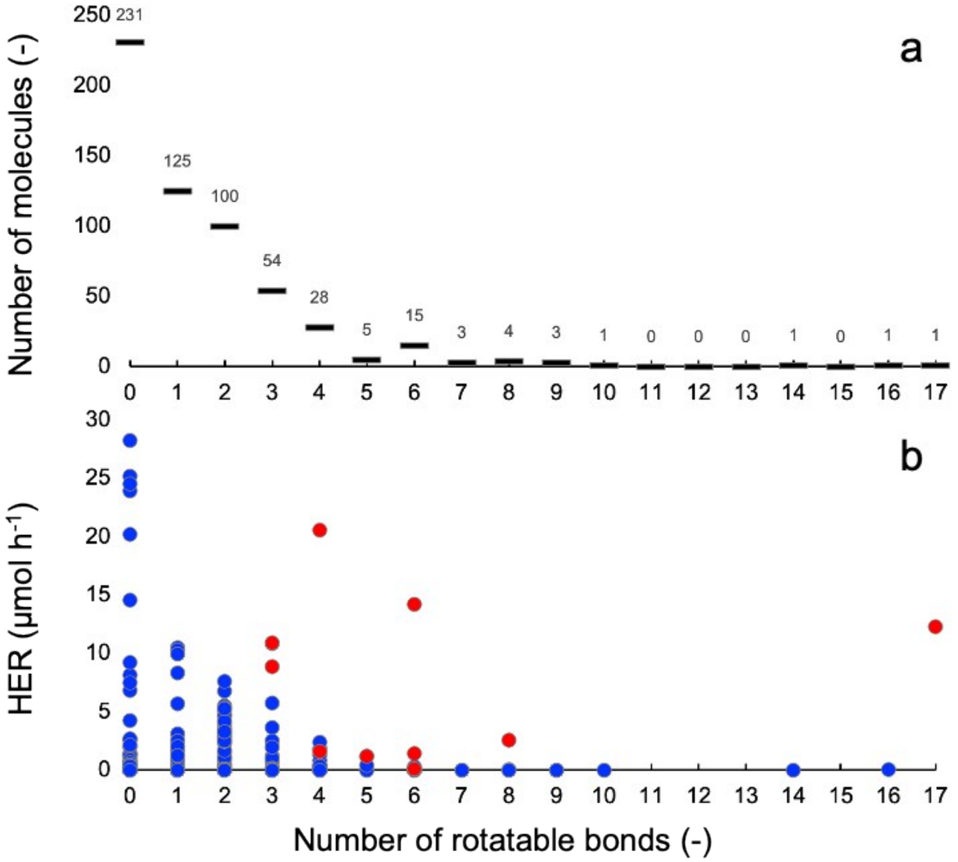

Figure S10. Distribution of the molecular photocatalyst library (572 molecules) as a function of the number of rotatable bonds within individual molecules: the number of molecules (a) or the hydrogen evolution rate (HER; b) is plotted against the number of rotatable bonds. For the ten molecules highlighted in red in (b), the effect of different conformers representing the same molecule on the resultant SOAP descriptors for the molecule was investigated. For each of these molecules, a conformer search was performed to screen all torsion angles that were not in a ring or with terminal hydrogen atoms. A Boltzmann jump search method was used, together with filtering the generated conformers by imposing the minimum variation in the root-mean-square of all sampled torsion angles being larger than 15°. After filtering, the five lowest-energy conformers were selected for representing the molecule for calculations of SOAP descriptors. The COMPASSIII force field was used. All conformer searches were performed in BIOVIA Materials Studio 2020. All the conformers thus generated were then geometry-optimized using B3LYP/6-3G*. Different 2D UMAP embeddings using the different conformers of the ten molecules are shown in Figure S11.

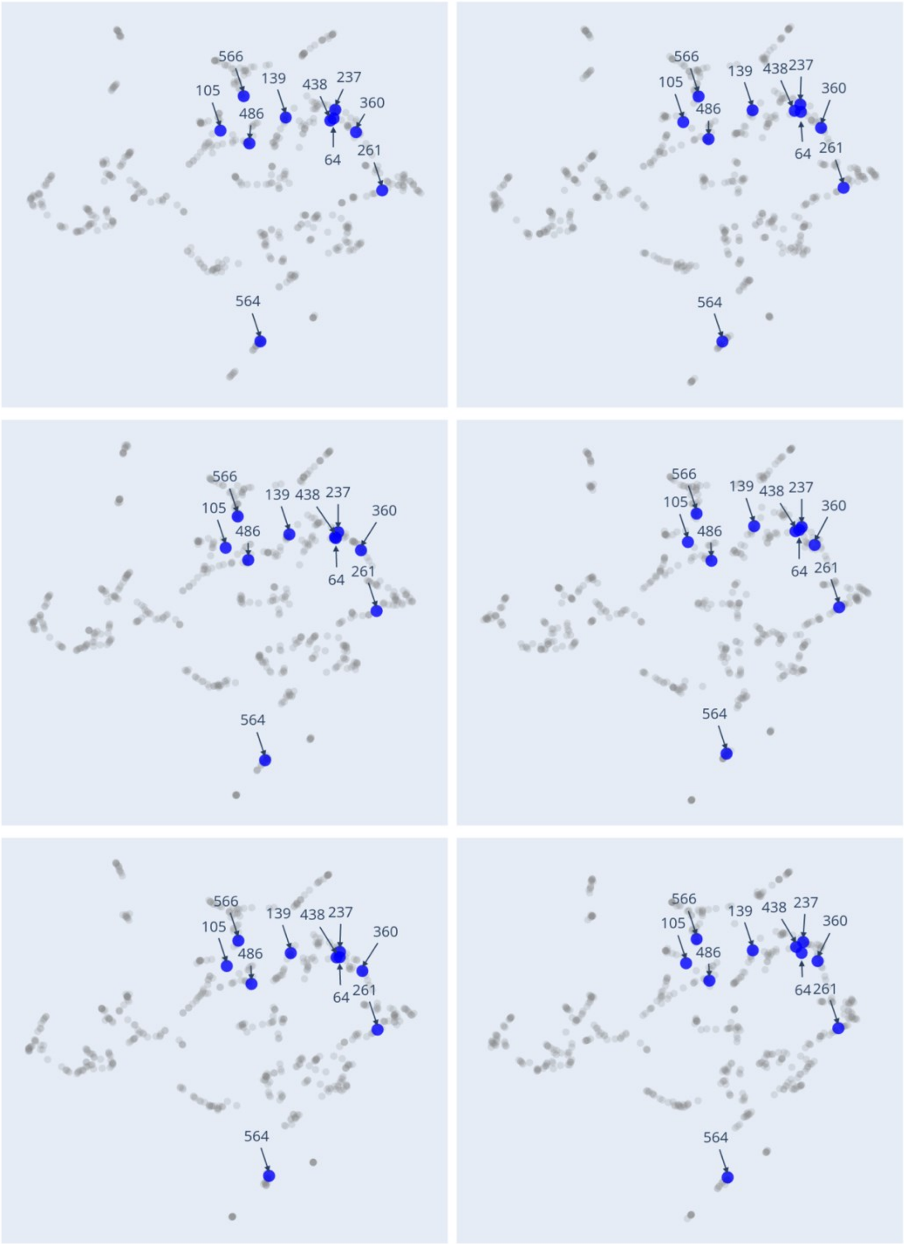

Figure S11. Aligned UMAP plots for the 572-molecule library, with the ten highlighted molecules (in blue) represented by the different conformers of them (see Figure S10) in the different plots; all the other molecules (in grey) were represented by the same molecular geometries across the different UMAP plots. The molecular geometries used throughout this work are shown in the UMAP plot in the top-left panel, while each of the other five panels shows a UMAP plot using a different local-minimum conformer for the ten highlighted molecules. This comparison shows that the 2D UMAP embeddings of the SOAP space of the 572 molecules are not markedly sensitive to the choice of molecular conformation for the ten highlighted molecules that are among the molecules in the library having a large number of rotatable bonds.

3.4. Machine learning with molecular descriptors

Table S1: Classifier threshold and model hyperparameters for each model after optimization.

Model	Binary Threshold	Binary hyperparameters	Ternary threshold	Ternary hyperparameters
GP	1.07	nu = 0.25<br>length_scale=251	(1.07, 12.5)	nu = 0.25<br>length_scale=251
RF	1.07	n_estimators = 10<br>max_features = 11<br>min_samples_split = 2<br>min_samples_leaf = 1<br>max_depth = 5	(1.07, 12.5)	n_estimators = 10<br>max_features = 11<br>min_samples_split = 2<br>min_samples_leaf = 1<br>max_depth = 5
GB-DT	1.07	learning_rate=0.001<br>n_estimators = 10000<br>min_samples_split = 73<br>min_samples_leaf = 1<br>max_depth = 5	(1.07, 12.5)	learning_rate=0.001<br>n_estimators = 10000<br>min_samples_split = 73<br>min_samples_leaf = 1<br>max_depth = 5
SVM	1.07	gamma = 1.945<br>C = 15.695	(1.07, 12.5)	gamma = 1.945<br>C = 15.695
MLP	1.07	epochs = 150<br>batch_size = 16<br>dropout = 0.05<br>dense_nodes = [16, 9]	(1.07, 12.5)	epochs = 150<br>batch_size = 16<br>dropout = 0.05<br>dense_nodes = [16, 9]
KNN	1.07	n_neighbors = 2	(1.07, 12.5)	n_neighbors = 2

Table S2. Area under the curve for the receiver operating characteristic curve (AUC ROC) and the precision-recall curve (AUC PR) for all the models across a 10-fold cross-validation.

Model	AUC ROC	AUC PR
KNN	0.749	0.417
GP	0.837	0.525
RF	0.852	0.606
GB-DT	0.875	0.629
SVM	0.871	0.563
MLP	0.691	0.354

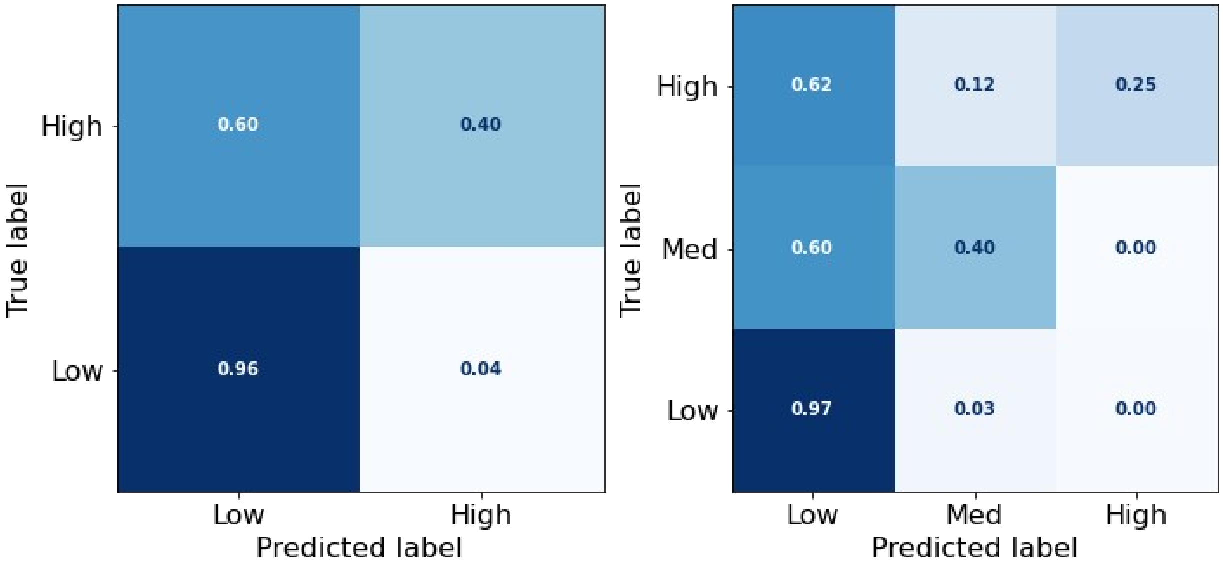

Figure S12. Confusion matrix for binary and ternary classifiers based on gradient boosted decision trees.

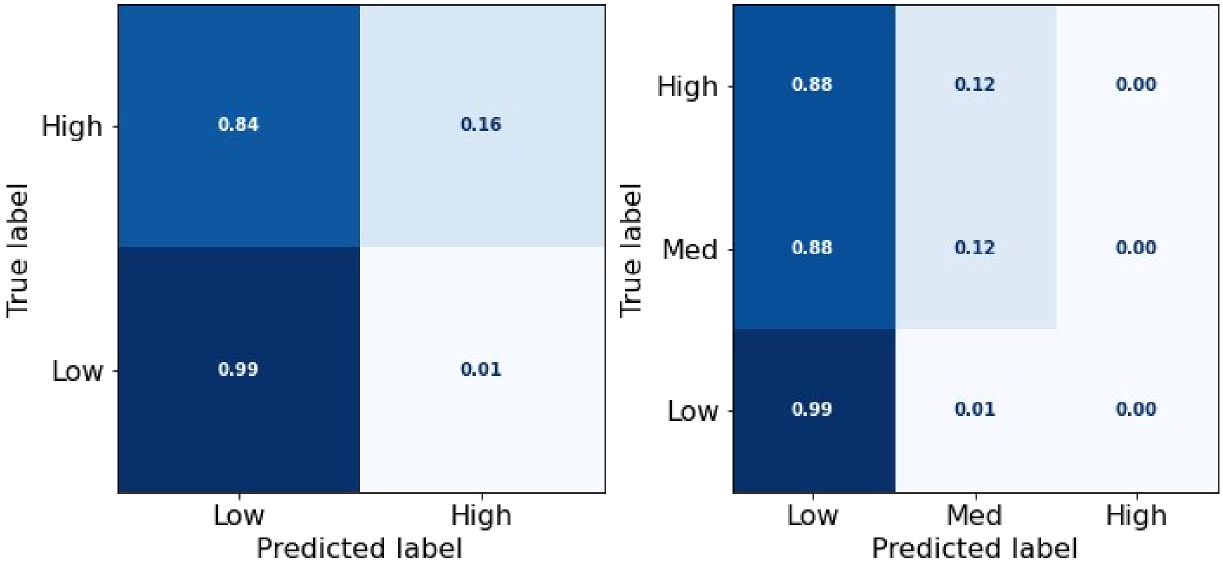

Figure S13. Confusion matrix for binary and ternary classifiers based on Gaussian processes.

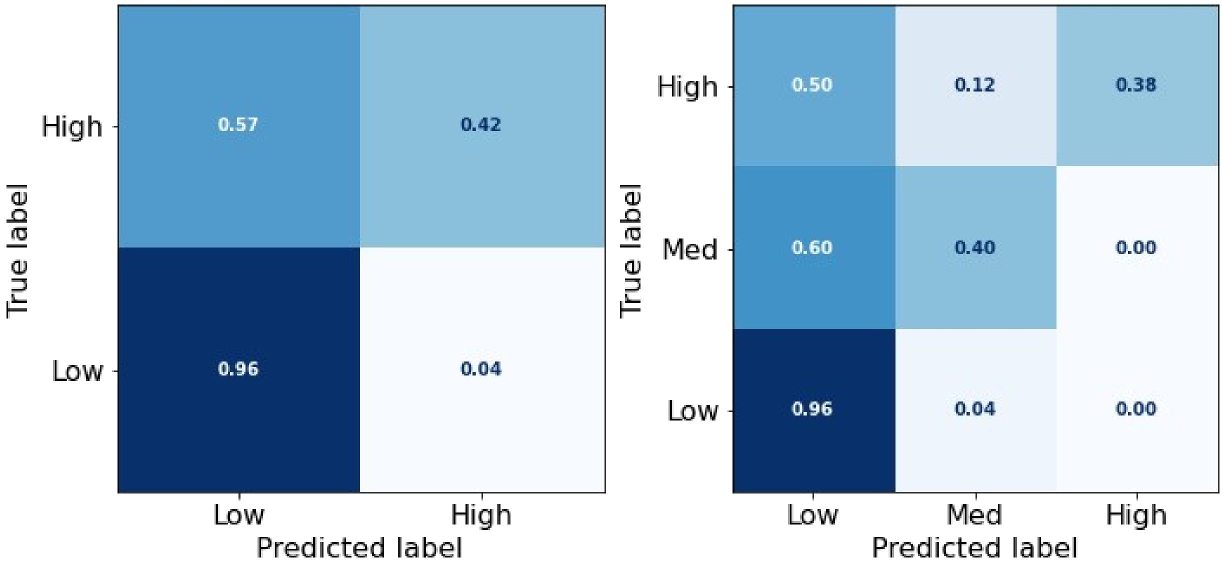

Figure S14. Confusion matrix for binary and ternary classifiers based on multilayer perceptrons.

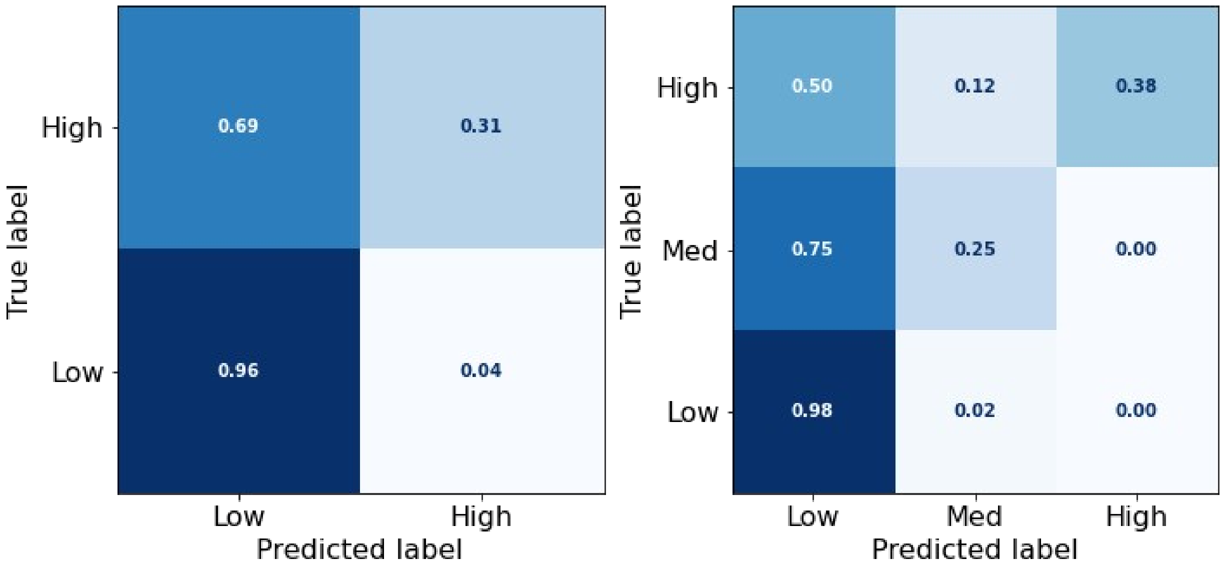

Figure S15. Confusion matrix for binary and ternary classifiers based on random forests.

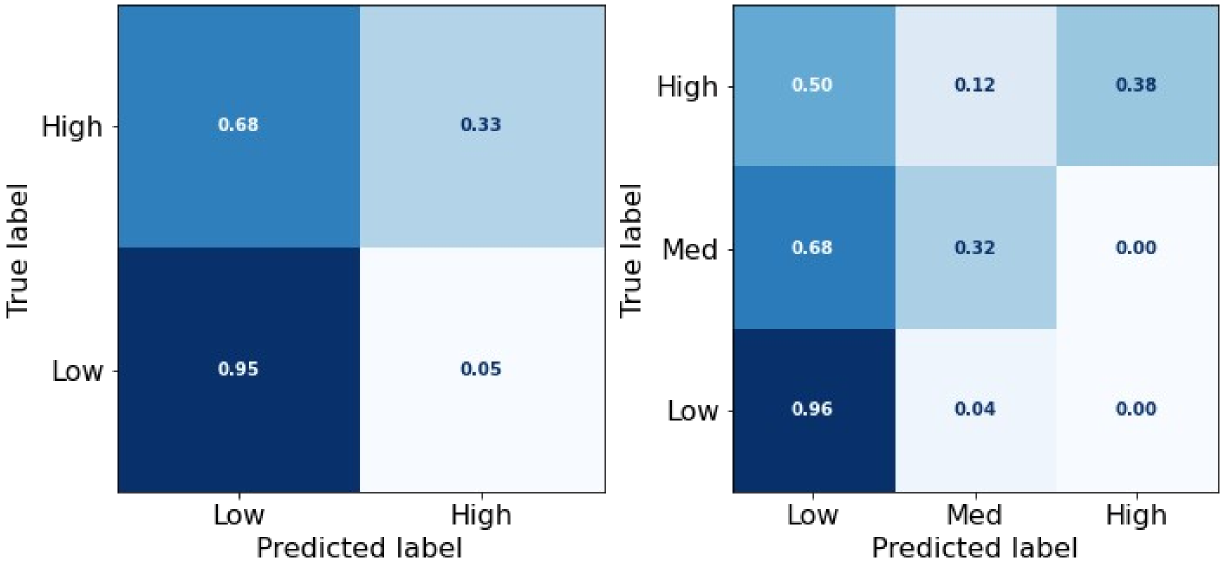

Figure S16. Confusion matrix for binary and ternary classifiers based on support vector machines.

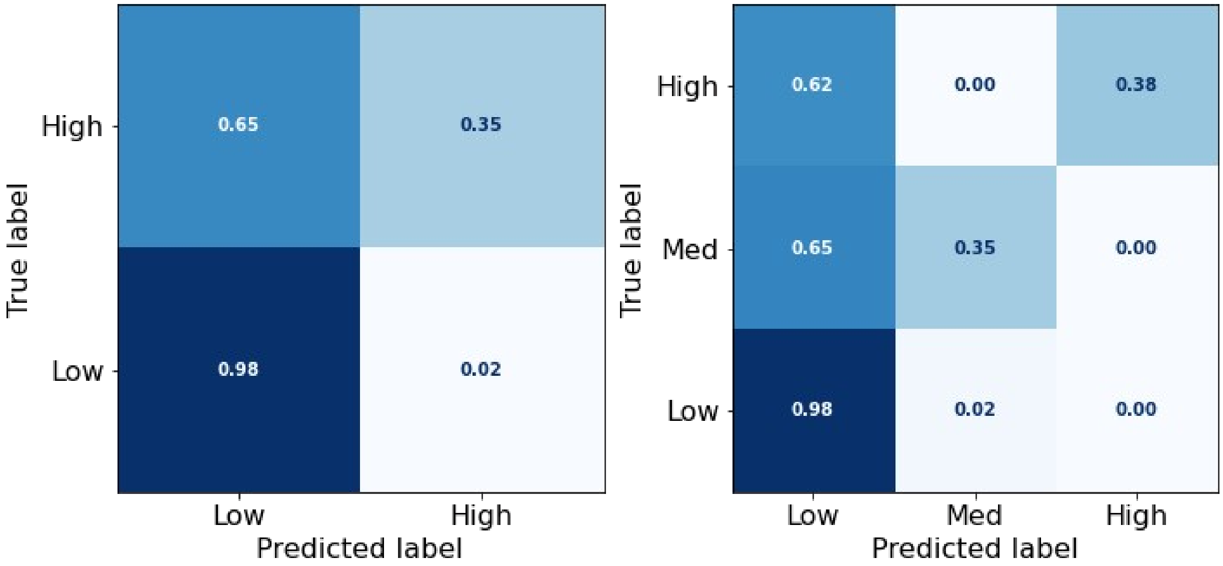

Figure S17. Confusion matrix for binary and ternary classifiers based on k-nearest neighbours.

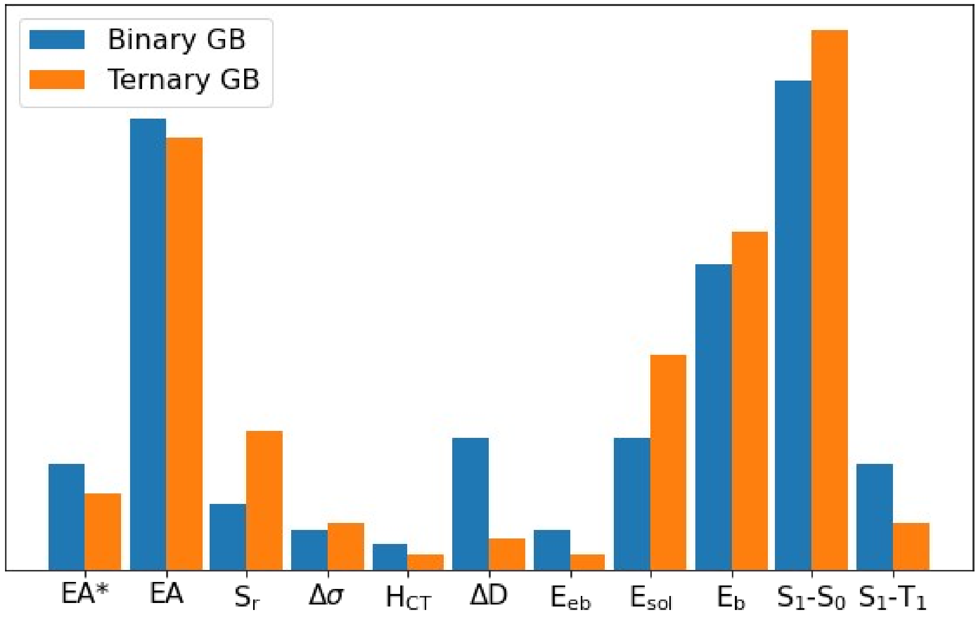

Figure S18. Plot of feature importance for the classifiers based on gradient boosted decision trees.

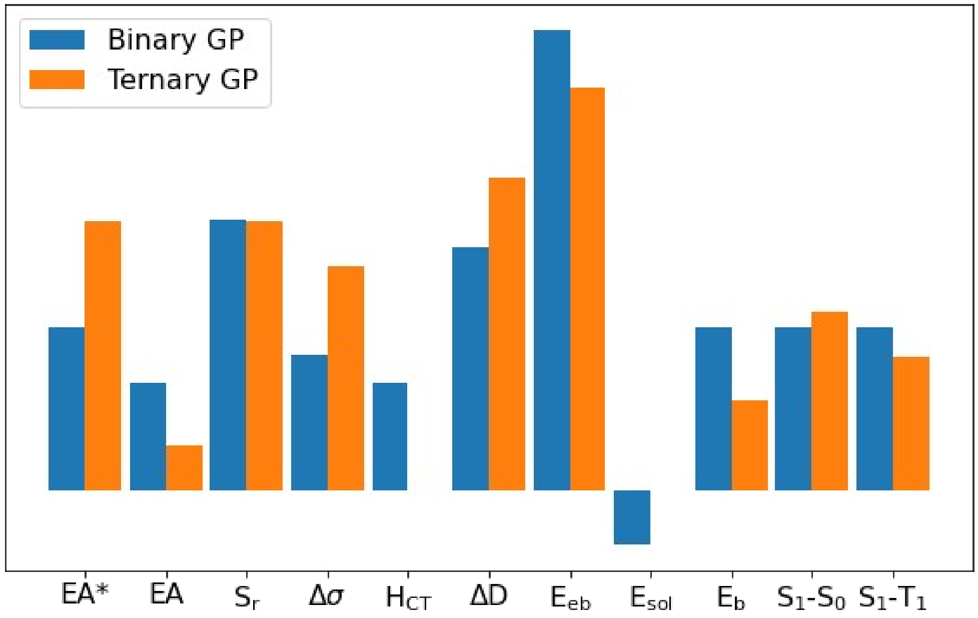

Figure S19. Plot of feature importance for the classifiers based on Gaussian processes.

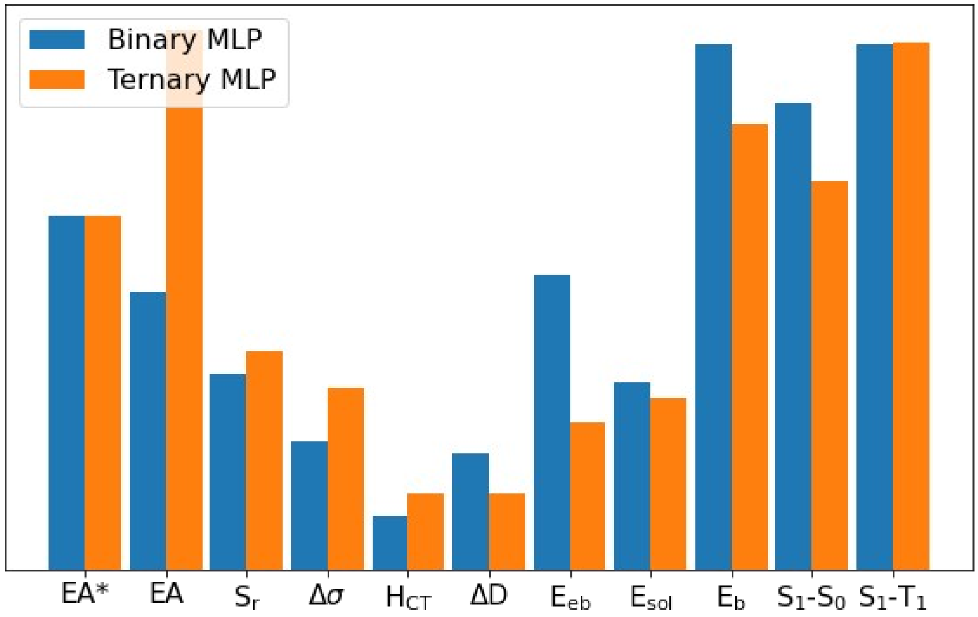

Figure S20. Plot of feature importance for the classifiers based on multilayer perceptrons.

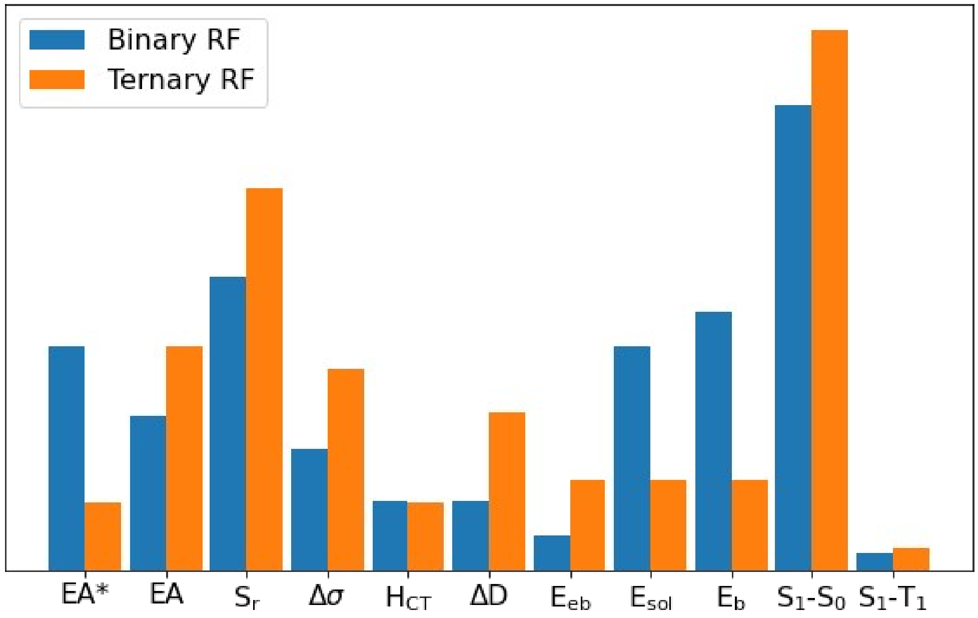

Figure S21. Plot of feature importance for the classifiers based on random forests.

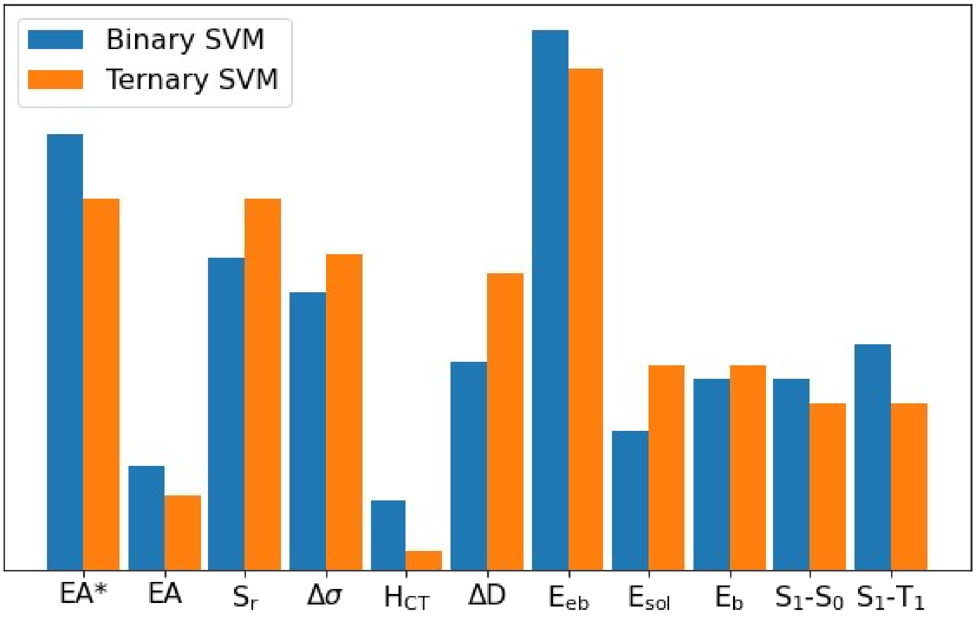

Figure S22. Plot of feature importance for the classifiers based on support vector machines.

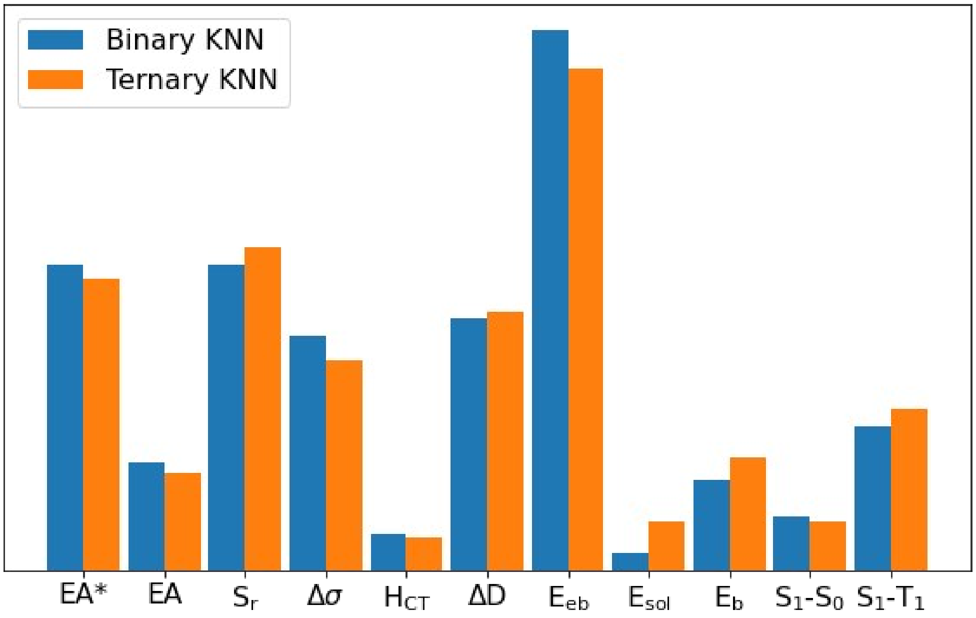

Figure S23. Plot of feature importance for the classifiers based on k-nearest neighbours.

3.5. Machine learning with molecular fingerprints and SOAP descriptors

Table S3. Binary and ternary classification metrics across models based molecular fingerprints or SOAP descriptors, obtained by 10-fold cross-validation procedures.

Representation	Model	Binary [a]			Ternary [a]	
		Accuracy	F1-score	MCC [f]	Accuracy	F1-score
Fingerprints [b]	KNN [d]	0.88	0.68	0.40	0.88	0.63
Fingerprints	SVM [e]	0.77	0.48	-0.04	0.77	0.34
SOAP [c]	KNN [d]	0.88	0.73	0.43	0.88	0.60
SOAP	SVM [e]	0.75	0.47	-0.06	0.84	0.32

[a] The class thresholds were the same as those in Table S1.

[b] Morgan fingerprints with a radius = 2, generated by RDKit (http://www.rdkit.org); similarity measure: Tanimoto index.

[c] SOAP descriptors with r = 6.0 Å, n = 8, l = 6, generated by DScribe (https://singroup.github.io/dscribe); similarity measure: regularized entropy match (REMatch) kernel. The similarity matrix for the 572 molecules used here is the same as the one used for Figure 2a in the main text.

[d] n_neighbors = 5; metric = precomputed.

[e] C = 15.6; metric = precomputed.

[f] The Matthews correlation coefficient, calculated directly from the binary confusion matrix (Figures S24–S27).

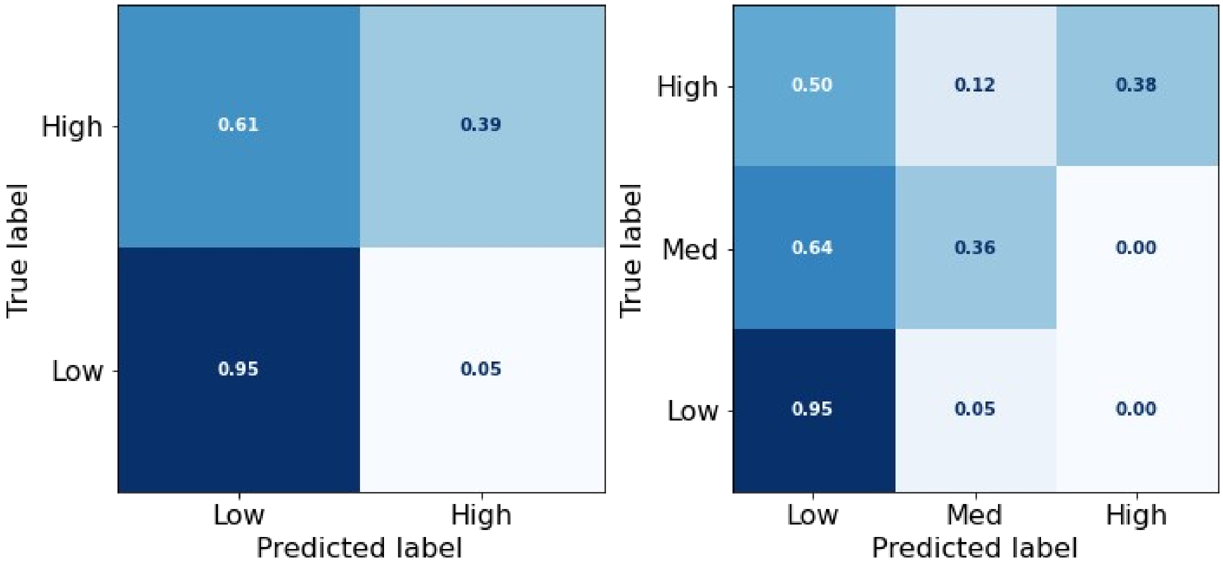

Figure S24. Confusion matrix for binary and ternary classifiers based on k-nearest neighbours and Morgan fingerprints.

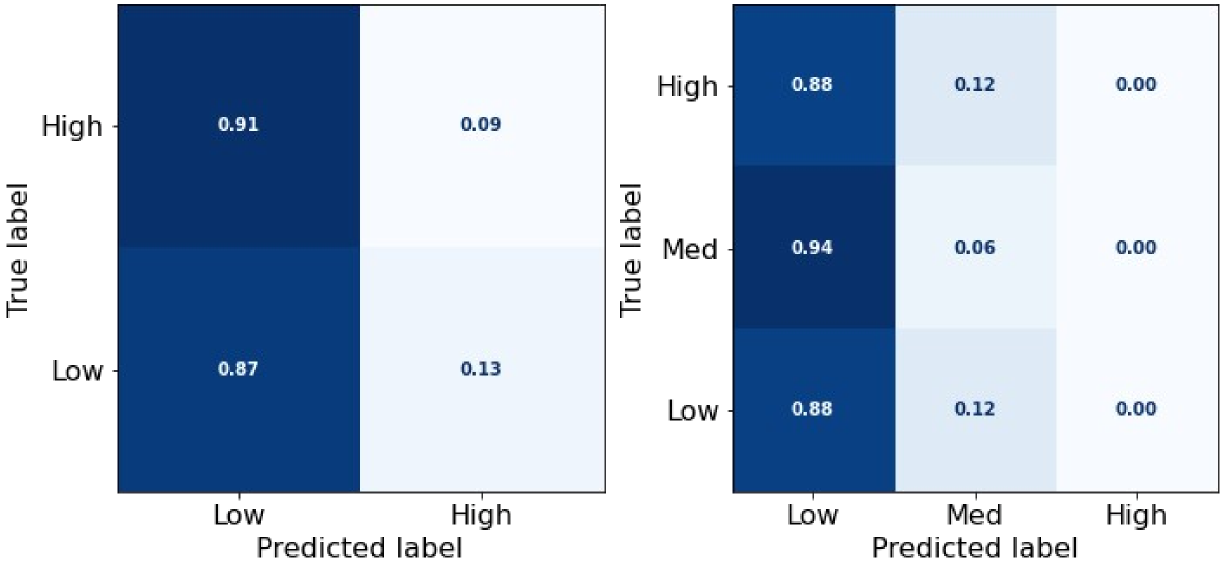

Figure S25. Confusion matrix for binary and ternary classifiers based on support vector machines and Morgan fingerprints.

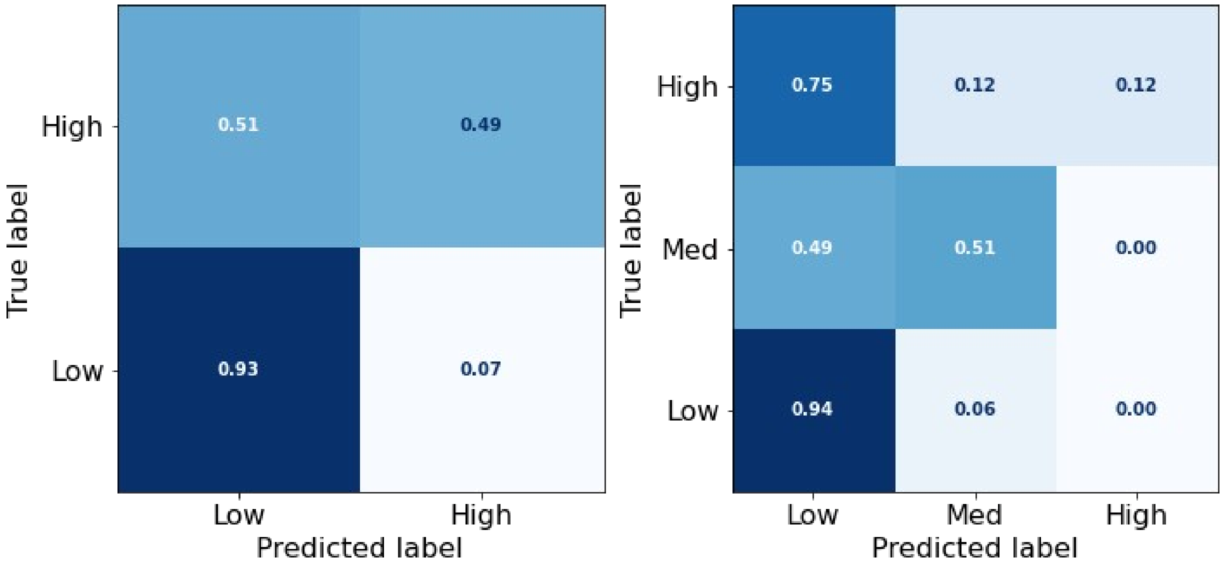

Figure S26. Confusion matrix for binary and ternary classifiers based on k-nearest neighbours and SOAP descriptors.

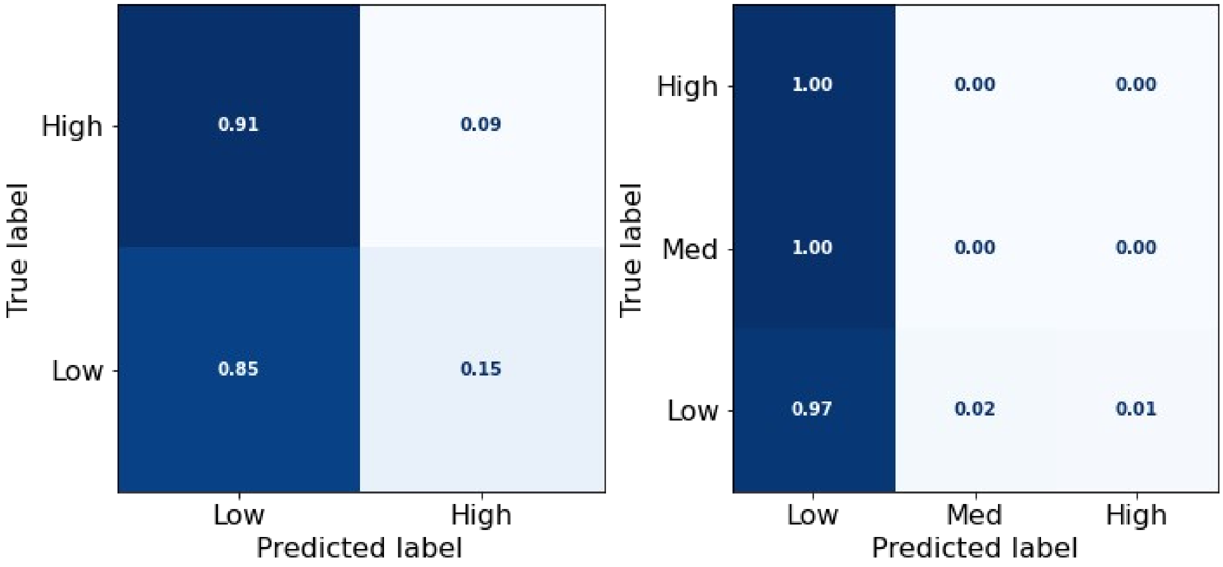

Figure S27. Confusion matrix for binary and ternary classifiers based on support vector machines and SOAP descriptors.

4. **Virtual experiments with the 572 molecules**

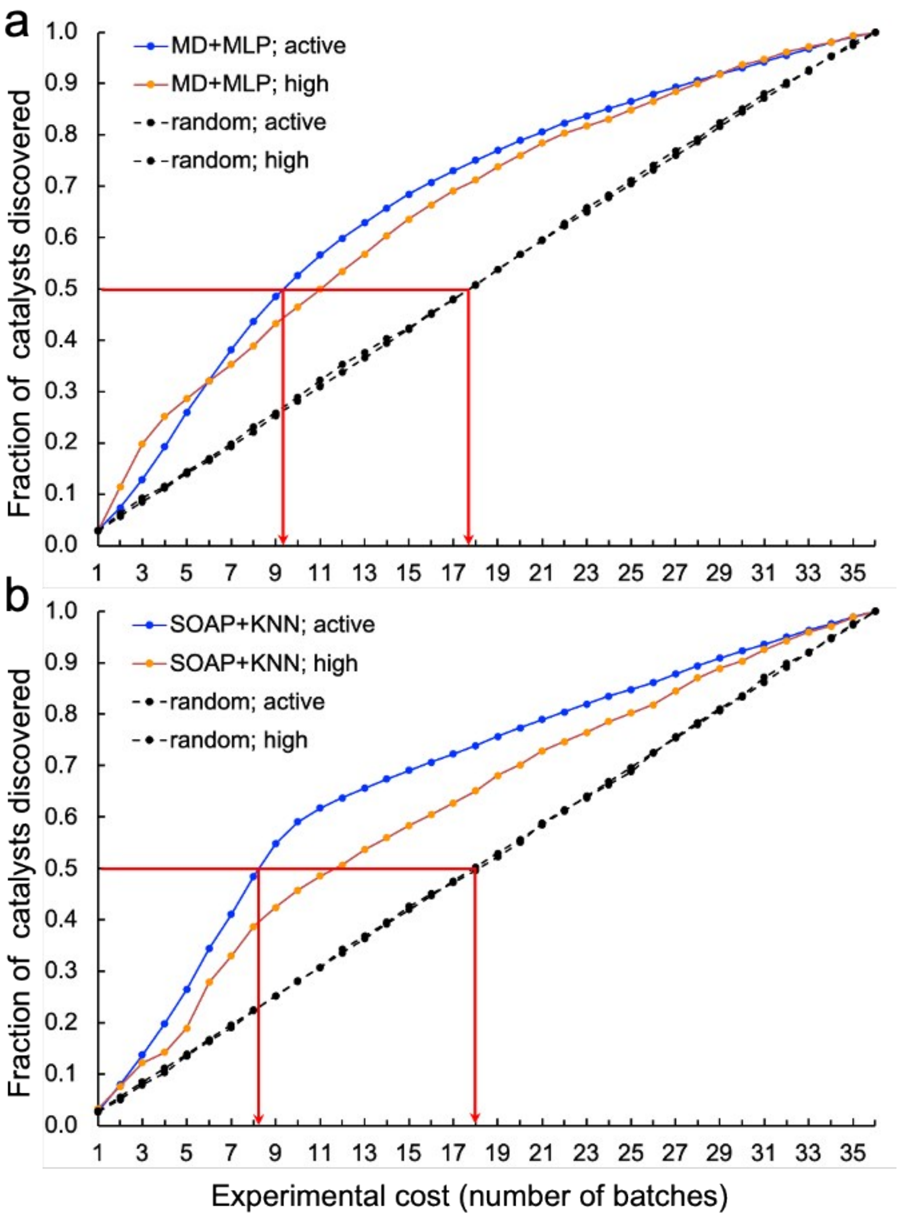

Figure S28. Virtual experiments comparing an adaptive ML approach and a random sampling approach: (**a**) Molecules were encoded by molecular descriptors (MD) and trained with MLP models; (**b**) molecules were encoded by SOAP descriptors and trained with KNN models. Active samples: HER > 1.07 µmol h^{-1}; high-activity samples: HER > 12.5 µmol h^{-1}. 200 *in silico* experiments, each with a different random starting point, were carried out for each case to obtain the average results shown. Each batch comprised 16 samples (molecules) – note that the experiments shown in the main text (Fig. 4) uses a batch size of 48, rather than 16. The performance increase attained for the smaller batch size of 16 is marginal.

5. Blind tests for 96 molecules, unseen by the models trained on the 572-molecule library

5.1. Molecules encoded by molecular optoelectronic descriptors

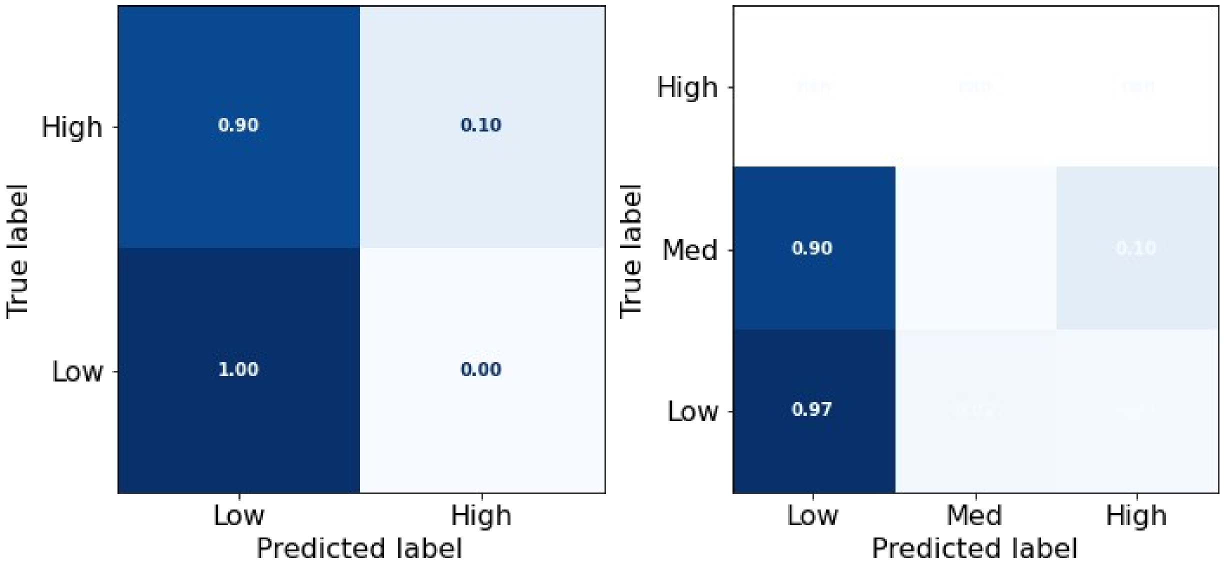

Figure S29. Confusion matrix for binary and ternary classifiers based on gradient boosted decision trees.

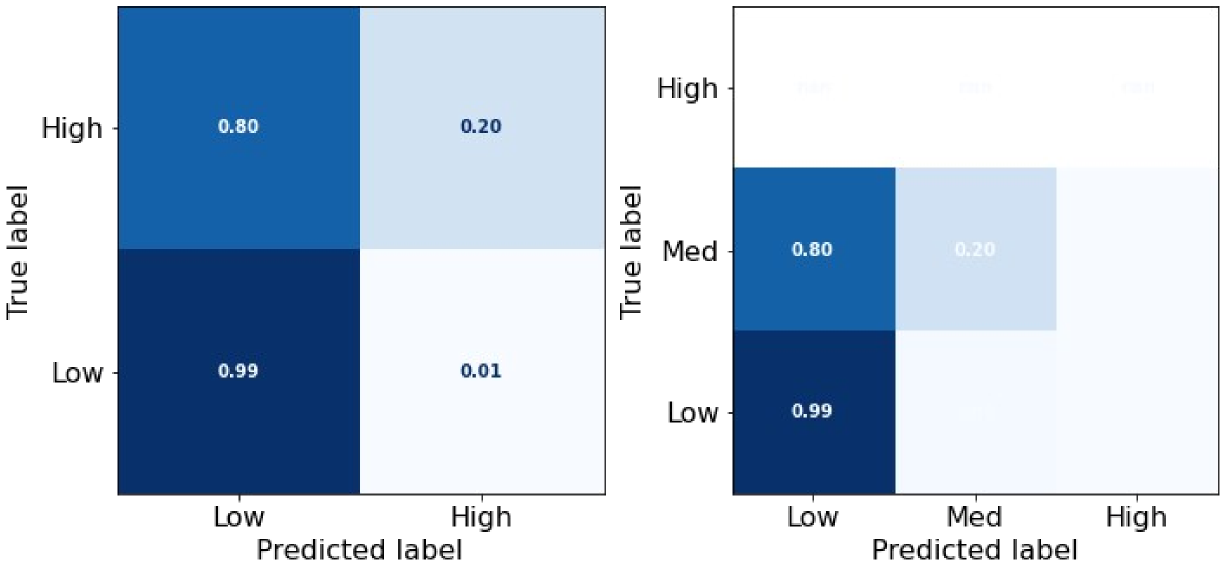

Figure S30. Confusion matrix for binary and ternary classifiers based on Gaussian processes.

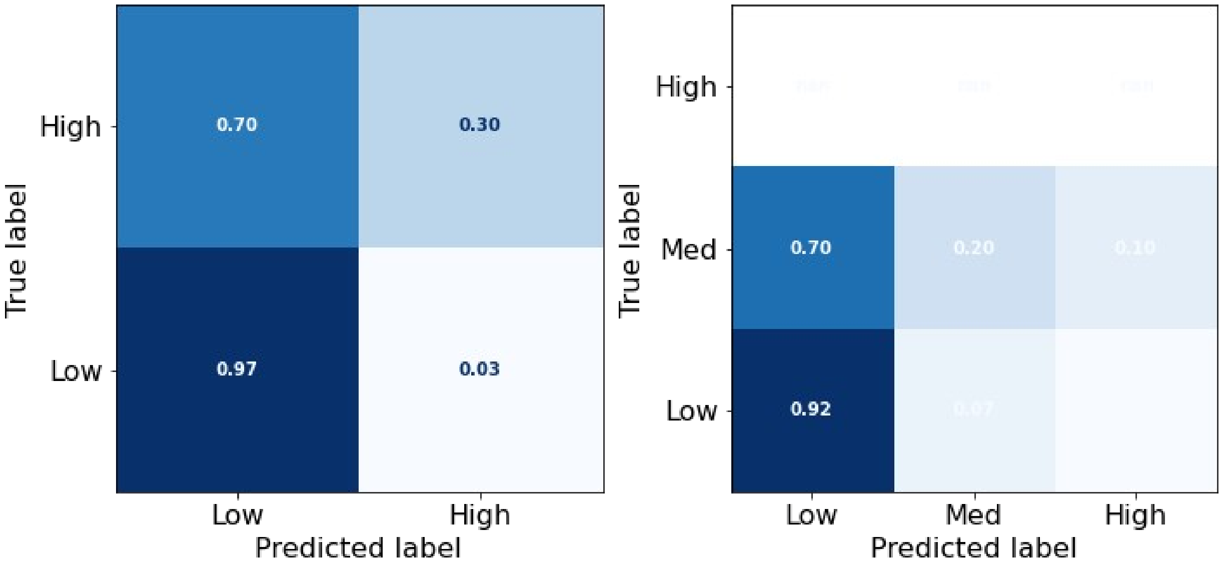

Figure S31. Confusion matrix for binary and ternary classifiers based on multilayer perceptrons.

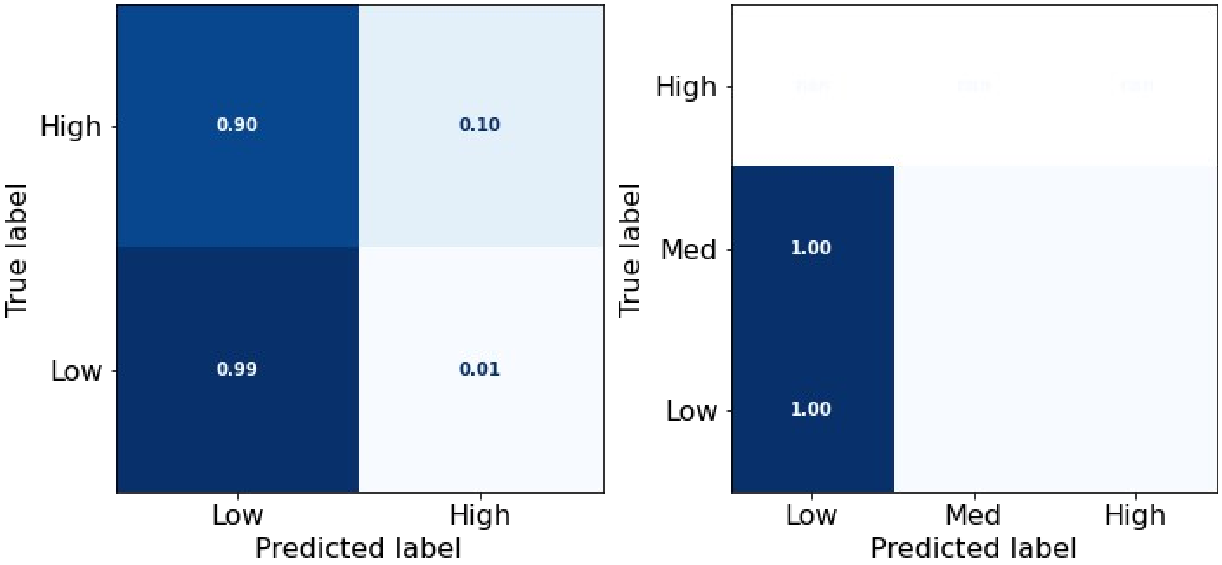

Figure S32. Confusion matrix for binary and ternary classifiers based on random forests.

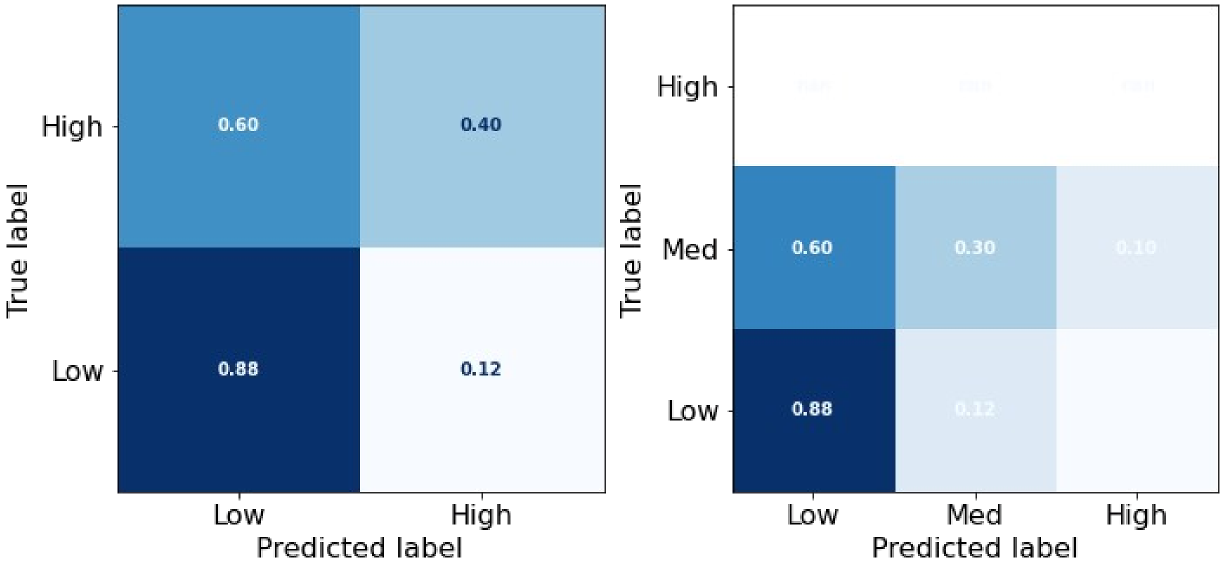

Figure S33. Confusion matrix for binary and ternary classifiers based on support vector machines.

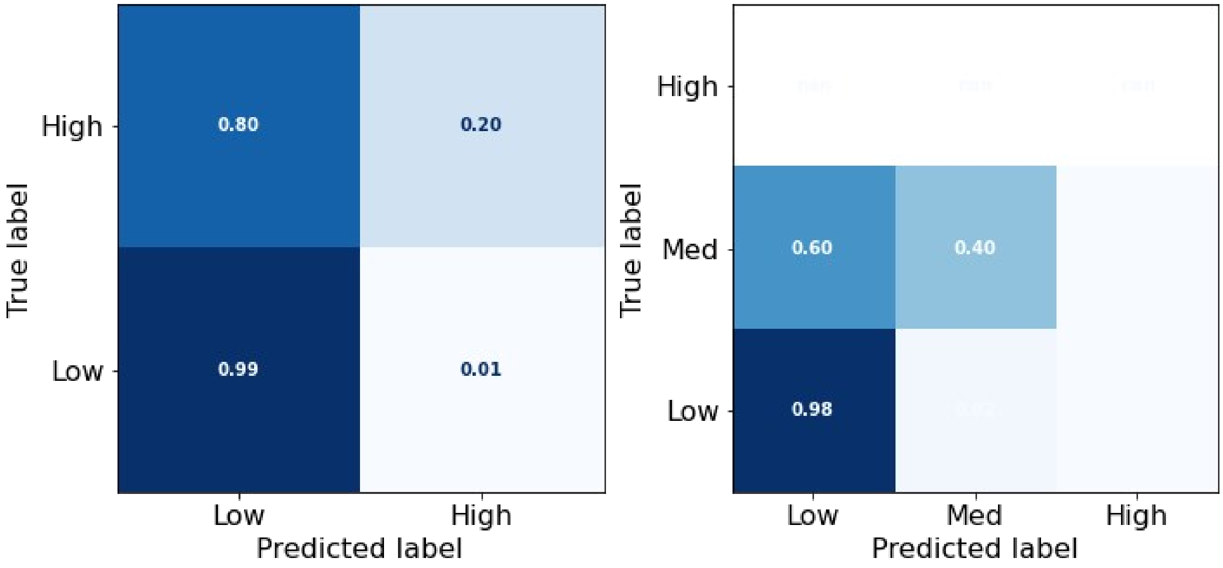

Figure S34. Confusion matrix for binary and ternary classifiers based on k-nearest neighbours.

5.2. Molecules encoded by SOAP descriptors

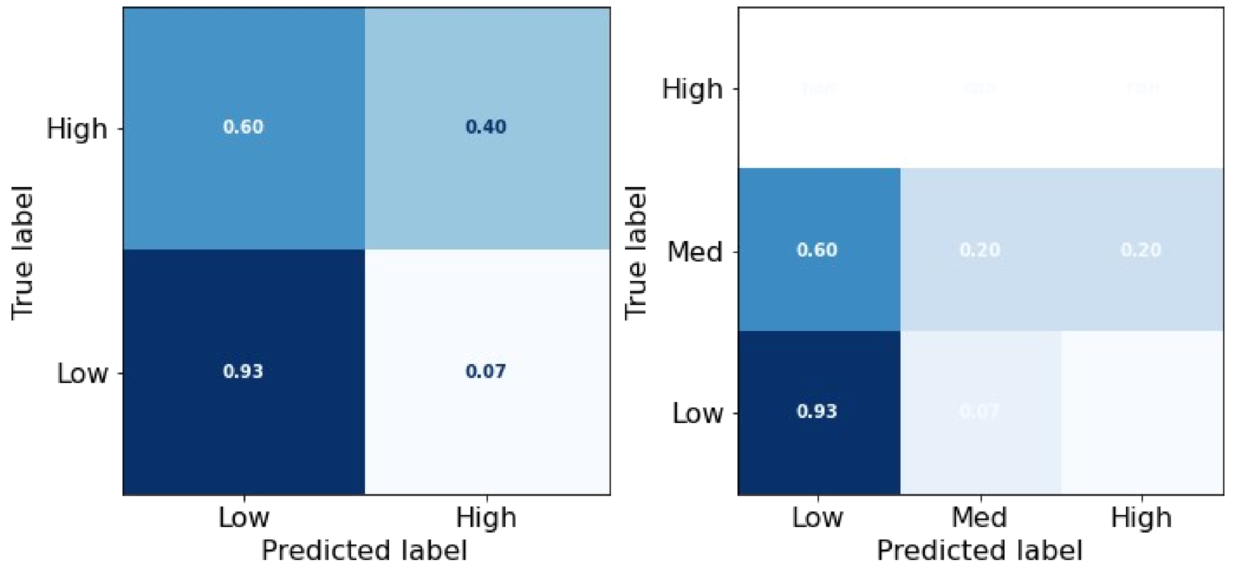

Figure S35. Confusion matrix for binary and ternary classifiers based on k-nearest neighbours and SOAP descriptors.

6. Experimental investigation of the effects of the amount of Pt cocatalyst and the choice of sacrificial agent on HERs

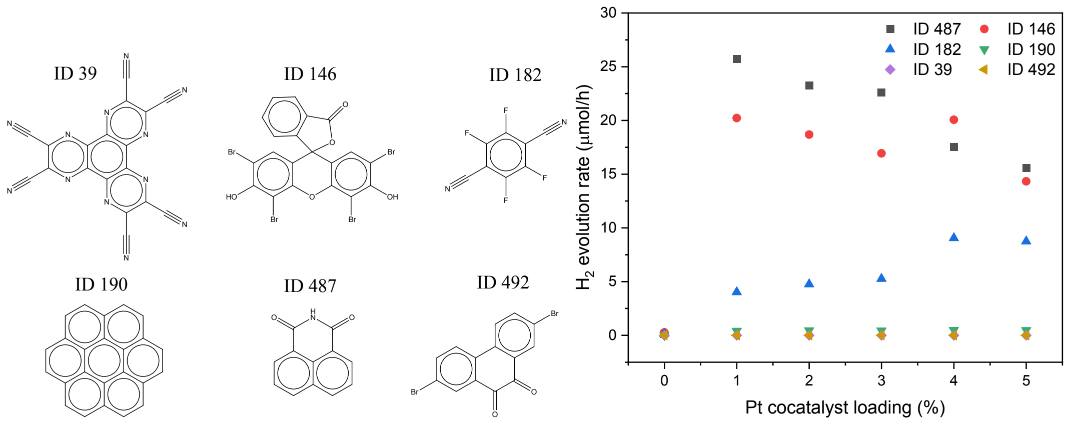

Figure S36. Measured HER as a function of the loading amount of Pt cocatalyst. Conditions: 5 mg molecular catalyst, triethylamine/methanol/H_2O (1:1:1 vol%) mixture, 0-5 wt% Pt (formed *in situ*), solar simulator irradiation (spectral range of source: 350 nm-1000 nm).

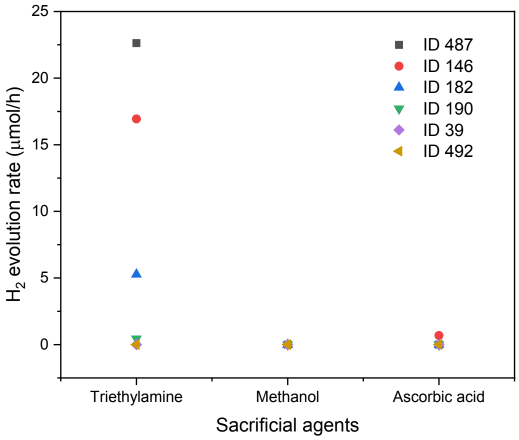

Figure S37. Measured HER as a function of the chosen sacrificial agent. 5 mg molecular catalyst, 3 wt% Pt (formed *in situ*), solar simulator irradiation (spectral range of source: 350 nm-1000 nm). Triethylamine conditions: Triethylamine/MEOH/H_2O (1:1:1 vol%) mixture; methanol conditions: MEOH/H_2O (1:2 vol%) mixture; ascorbic acid condition: 0.1 M ascorbic acid in MEOH/H_2O (1:2 vol%) mixture.

7. References

1. Li, X.; Melissen, S. T. A. G.; Le Bahers, T.; Sautet, P.; Masters, A. F.; Steinmann, S. N.; Maschmeyer, T., Shining Light on Carbon Nitrides: Leveraging Temperature To Understand Optical Gap Variations. *Chem. Mater.* **2018,** *30* (13), 4253-4262.
2. Ren, S.; Bojdys, M. J.; Dawson, R.; Laybourn, A.; Khimyak, Y. Z.; Adams, D. J.; Cooper, A. I., Porous, Fluorescent, Covalent Triazine-Based Frameworks Via Room-Temperature and Microwave-Assisted Synthesis. *Adv. Mater.* **2012,** *24* (17), 2357-2361.
3. O'Boyle, N. M.; Banck, M.; James, C. A.; Morley, C.; Vandermeersch, T.; Hutchison, G. R., Open Babel: An open chemical toolbox. *J. Cheminformatics* **2011,** *3* (1), 33.
4. Morris, G. M.; Huey, R.; Lindstrom, W.; Sanner, M. F.; Belew, R. K.; Goodsell, D. S.; Olson, A. J., AutoDock4 and AutoDockTools4: Automated docking with selective receptor flexibility. *J. Comput. Chem.* **2009,** *30* (16), 2785-2791.
5. Grimme, S.; Bannwarth, C.; Shushkov, P., A Robust and Accurate Tight-Binding Quantum Chemical Method for Structures, Vibrational Frequencies, and Noncovalent Interactions of Large Molecular Systems Parametrized for All spd-Block Elements (Z = 1–86). *J. Chem. Theory Comput.* **2017,** *13* (5), 1989-2009.
6. Peach, M. J. G.; Benfield, P.; Helgaker, T.; Tozer, D. J., Excitation energies in density functional theory: An evaluation and a diagnostic test. *J. Chem. Phys.* **2008,** *128* (4), 044118.
